# *E/Z* Isomerism of the Potential Antiepileptic Drug GIZH-298

**DOI:** 10.3390/molecules31111872

**Published:** 2026-05-29

**Authors:** Grigory V. Mokrov, Svetlana A. Litvinova, Valentina E. Biryukova, Tatiana A. Voronina, Sergey V. Shorunov, Alexey G. Rebeko, Tatiana Yu. Vorobyova, Liubov N. Grushevskaya, Maria S. Sergeeva, Dmitry N. Kuznetsov, Oxana Yu. Kravtsova, Ekaterina A. Nikitina, Natalia A. Gladysheva, Aliy K. Zhanataev, Andrey D. Durnev, Timofey V. Losev, Ksenia M. Malashkeevich, Michael G. Medvedev, Marat M. Islamov, Konstantin A. Lyssenko, Vladimir L. Dorofeev

**Affiliations:** 1 Department of Medicinal Chemistry, Federal Research Center for Innovator and Emerging Biomedical and Pharmaceutical Technologies, Baltiyskaya 8, 125315 Moscow, Russia; shorunov_sv@academpharm.ru (S.V.S.); rebeko_ag@academpharm.ru (A.G.R.); vorobeva_tyu@academpharm.ru (T.Y.V.); 2 Department of Neuropsychopharmacology, Federal Research Center for Innovator and Emerging Biomedical and Pharmaceutical Technologies, Baltiyskaya 8, 125315 Moscow, Russia; litvinova_sa@academpharm.ru (S.A.L.); voronina_ta@academpharm.ru (T.A.V.); nikitina_ea@academpharm.ru (E.A.N.); gladysheva_na@academpharm.ru (N.A.G.); 3 Laboratory of Standardization and Quality Control, Federal Research Center for Innovator and Emerging Biomedical and Pharmaceutical Technologies, Baltiyskaya 8, 125315 Moscow, Russia; grushevskaya_ln@academpharm.ru (L.N.G.); sergeeva_ms@academpharm.ru (M.S.S.); kuznetsov_dn@academpharm.ru (D.N.K.); dorofeev_vl@academpharm.ru (V.L.D.); 4 Pharmacokinetics Laboratory, Federal Research Center for Innovator and Emerging Biomedical and Pharmaceutical Technologies, Baltiyskaya 8, 125315 Moscow, Russia; kravtsova.oxan@mail.ru; 5 Department of Toxicology, Federal Research Center for Innovator and Emerging Biomedical and Pharmaceutical Technologies, Baltiyskaya 8, 125315 Moscow, Russia; zhanataev_ak@academpharm.ru (A.K.Z.); durnev_ad@academpharm.ru (A.D.D.); 6 Group of Theoretical Chemistry, Zelinsky Institute of Organic Chemistry, Russian Academy of Sciences, Leninsky Prospekt 47, 119991 Moscow, Russia; losev@ioc.ac.ru (T.V.L.); kseniamalaskeevic@gmail.com (K.M.M.); medvedev.m.g@gmail.com (M.G.M.); 7 Faculty of Chemistry, Lomonosov Moscow State University, Leninskie Gory 1/3, 119991 Moscow, Russia; marat.islamov@chemistry.msu.ru (M.M.I.); klyssenko@gmail.com (K.A.L.); 8 Federal Research Center for Innovator and Emerging Biomedical and Pharmaceutical Technologies, Baltiyskaya 8, 125315 Moscow, Russia

**Keywords:** GIZH-298, *E/Z*-isomerism, anticonvulsant activity, oximes, 4-benzoylpyridine oximes

## Abstract

The synthesis, purification, and comprehensive characterization of the *E*- and *Z*-isomers of the prospective anticonvulsant compound GIZH-298 were carried out. It was demonstrated that both isomers could be obtained in a pure form by isolating the corresponding oximes at the initial stage of synthesis. The *E*- and *Z*-isomers exhibit pronounced differences in their physicochemical characteristics, spectral features, and pharmacological profiles. *Z*-GIZH-298 was found to be photosensitive, undergoing *Z-E* isomerization upon irradiation, as confirmed by both theoretical analysis and experimental observations. Additionally, partial conversion to the *E*-form occurs during prolonged reflux of *Z*-GIZH-298 in polar solvents, whereas the compound remains stable in aqueous and acidic media at ambient temperature. In the maximal electroshock assay, both isomers demonstrated anticonvulsant activity, with *Z*-GIZH-298 showing substantially higher efficacy than its *E*-counterpart. These findings highlight the need to account for potential *E*-isomer formation when considering further development of *Z*-GIZH-298 as a promising anticonvulsant agent.

## 1. Introduction

According to the estimates from the World Health Organization and epidemiological surveys, approximately 50–70 million people worldwide are affected by epilepsy. It is the second most common neurological disorder after stroke [1,2]. Despite substantial progress in epilepsy research, nearly 30% of patients continue to experience uncontrolled and debilitating seizures. In the international classification of epilepsies, epileptic syndromes, and related disorders, epilepsy is not treated as a single disease characterized only by different seizure types; rather, it is divided into distinct clinical forms—approximately 40 epileptic syndromes—each defined by specific electroclinical features and associated with its own prognosis and therapeutic strategies [3]. Even when adequate seizure control is achieved, many available antiepileptic drugs (AEDs) often lead to adverse side effects—including anxiety, depression, memory impairment, reduced attention, and drowsiness—due to their nonspecific actions on the central nervous system [4,5].

**Figure 1:**
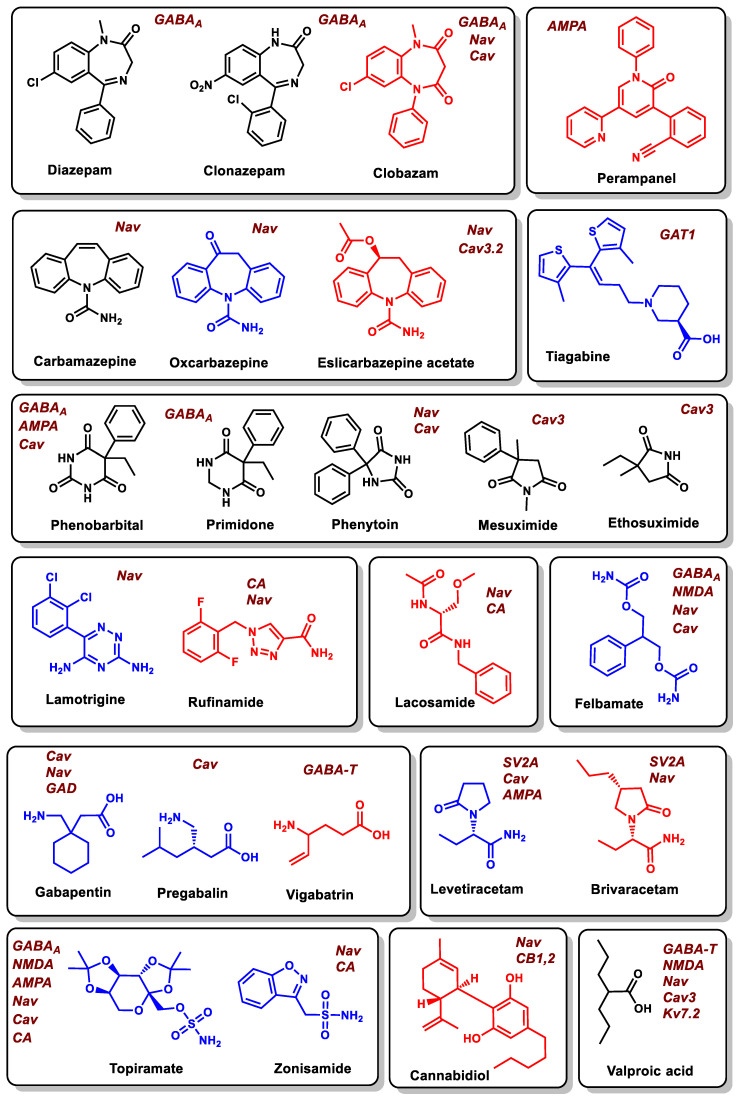
Main FDA-approved antiepileptic drugs (AEDs). First-generation AEDs are shown in black, second-generation in blue, and third-generation in red. Proposed biomolecular targets are indicated next to each drug.

According to the U.S. Food and Drug Administration, nearly 30 AEDs from diverse pharmacological classes are currently used in clinical practice (Figure 1). The most widely used agents include benzodiazepine derivatives (first-generation diazepam and clonazepam; third-generation clobazam); the triaromatic pyridinone derivative perampanel (third generation); dibenzoazepine derivatives (first-generation carbamazepine; second-generation oxcarbazepine; third-generation eslicarbazepine acetate); the nipecotic acid derivative tiagabine (second generation); cyclic amide, diamide, and diimide derivatives (first-generation phenobarbital, primidone, phenytoin, mesuximide, ethosuximide); triazaheterocycle derivatives (second-generation lamotrigine; third-generation rufinamide); non-cyclic amides (second-generation felbamate; third-generation lacosamide); GABA derivatives (second-generation gabapentin and pregabalin; third-generation vigabatrin); 2-pyrrolidone derivatives (second-generation levetiracetam; third-generation brivaracetam); sulfonamide compounds (second-generation topiramate and zonisamide); as well as cannabidiol (third generation) and valproic acid (first generation). Among these, several third-generation drugs—such as brivaracetam, lacosamide, and perampanel—have been introduced into epilepsy pharmacotherapy in recent years. However, despite their improved tolerability and efficacy in certain forms of the disease, they have not solved the challenge of pharmacoresistant epilepsy [6]. As a result, the development of new therapeutic options for epilepsy remains an urgent and ongoing priority.

At the Federal Research Center for Innovators and Emerging Biomedical and Pharmaceutical Technologies, a novel compound designated *Z*-GIZH-298 (the oxalate salt of 4-benzoylpyridine *Z*-*O*-(2-morpholinoethyl)oxime) is being developed as a promising antiepileptic agent. This compound has demonstrated robust efficacy in several animal tests and models of epilepsy, including maximal electroshock (mice and rats), partial secondarily generalized seizures in rats with chronic cobalt-induced epileptogenic foci, as well as status epilepticus induced by homocysteine thialactone in rats with a chronic epilepsy model, and in a genetic model of epilepsy (Krushinsky–Molodkina rats) [7,8].

Considering the mechanistic data on the reference compounds that guided the design of the benzoylpyridine derivative series, along with the results from molecular docking studies, an in vitro assessment of the potential interactions of *Z*-GIZH-298 with a panel of receptors and ion channels was performed (partially unpublished data; Table 1). The panel included dopamine receptors (D1, D2, and D3), the AMPA receptor, serotonin receptors (5-HT_1A_ and 5-HT_2C_), L-type Ca^2+^ channels (dihydropyridine, diltiazem, and verapamil binding sites), N-type Ca^2+^ channels, the voltage-gated Na^+^ channel, the dopamine transporter, and the serotonin transporter. Affinity profiling was conducted using radioligand binding assays, with the corresponding radioligands listed in Table 1 [9,10]. For *Z*-GIZH-298, IC_50_ values for all tested biomolecular targets exceeded 10 μM. Nevertheless, this does not exclude a potential contribution of these targets to the compound’s mechanism of action at higher concentrations.

### 1.1. Ex Vivo Analysis of Putative Biomolecular Targets Involved in the Anticonvulsant Mechanism of Z-GIZH-298

To investigate the potential involvement of glutamate, dopamine, and serotonin receptors in the anticonvulsant action of *Z*-GIZH-298, receptor profiles in the rat brain (NMDA (*N*-methyl-D-aspartate receptors), mGluII/mGluR2/3, 5-HT_2A_, and D2 receptors) and in mice (D2 receptors) were analyzed following tonic-seizures induced by maximal electroshock (MES) [9,10]. MES changed the balance of NMDA/mGluII (mGluR2/3) receptor density in the brain and decreased D2 receptor density, while quantitative parameters of 5-HT_2A_ receptors remained unchanged. Administration of *Z*-GIZH-298 abolished seizure manifestations but did not reverse MES-induced changes in NMDA and mGluR2/3 receptor levels, nor did it affect these receptors under baseline conditions, but it counteracted the MES-induced reduction in striatal D2 receptor levels.

Additionally, in non-MES-exposed mice, *Z*-GIZH-298 increased D2 receptor density in the striatum. These results suggest that the anticonvulsant effects of *Z*-GIZH-298 are linked to the restoration of striatal D2 receptor levels, supporting the hypothesis that D2 receptors may represent a functional molecular target of the compound [9,10].

The involvement of ERK1/2 (extracellular signal-regulated kinase ½) kinase in the anticonvulsant mechanism of *Z*-GIZH-298 was also evaluated. ERK1/2 are extracellular signal-regulated kinases that play key roles in neuronal activity and synaptic plasticity, and their functional alterations have been documented under seizure conditions. MES exposure in mice increased phosphorylation of ERK1/2 and synapsin I in the striatum. Post-MES administration of *Z*-GIZH-298 reduced phospho-ERK1/2 and phospho-synapsin I levels, correlating with decreased seizure severity. Furthermore, *Z*-GIZH-298 suppressed ERK1/2 phosphorylation in human SH-SY5Y neuroblastoma cells at therapeutically relevant concentrations. These findings indicate that modulation of ERK1/2 kinase activity may constitute a component of the anticonvulsant mechanism of *Z*-GIZH-298 [10].

### 1.2. Neurochemical Effects of Z-GIZH-298 on Monoamine Levels and Their Metabolites in Rat Brain Structures

The effects of *Z*-GIZH-298 on monoamine neurotransmitters and their metabolites, as well as inhibitory and excitatory amino acids, were evaluated in the brains of rats and mice using high-performance liquid chromatography (HPLC) with electrochemical detection [7,11,12]. Following generalized tonic-clonic seizures induced by MES, intraperitoneal injection (i.p.) administration of *Z*-GIZH-298 prevented MES-induced hyperactivity of the dopaminergic system and the concomitant decrease in striatal norepinephrine levels. The compound also restored the disrupted GABA/glutamate balance and normalized levels of inhibitory amino acids—including taurine, glycine, and GABA—in the hypothalamus. These results suggest that the anticonvulsant effects of *Z*-GIZH-298 in the MES model involve modulation of noradrenergic and dopaminergic neurotransmission, ERK1/2 kinase activity in the striatum, and restoration of inhibitory amino acid balance in the hypothalamus.

### 1.3. Preclinical Development of Z-GIZH-298 as a Potential Anticonvulsant

The compound *Z*-GIZH-298 is currently completing the full preclinical development cycle at the relevant divisions of the Federal Research Center for Innovator and Emerging Biomedical and Pharmaceutical Technologies. A summary of the available results to date is presented in Table 2. It should be noted that, in addition to its primary pharmacological activity, *Z*-GIZH-298 has been found to possess anxiolytic [13] and analgesic properties [14].

**Scheme 1:**
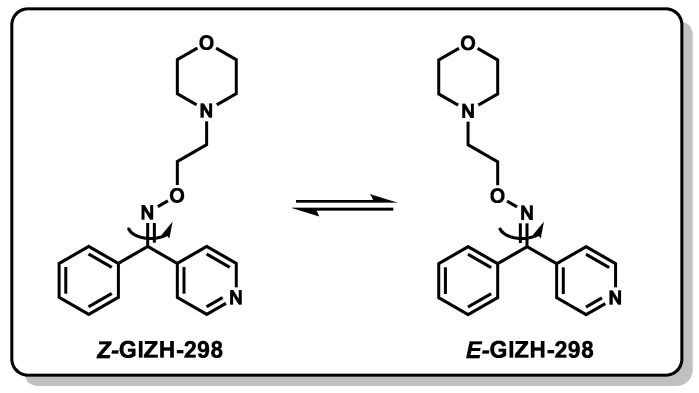
Structures of GIZH-298 isomers.

Since GIZH-298 is a distinct geometric isomer of a substituted oxime, it is important to evaluate its potential for *E/Z*-isomerism (Scheme 1). The literature indicates that substituted asymmetric oximes can partially interconvert between geometric isomers under specific conditions, such as irradiation or heating. For example, UV irradiation induces the photoisomerization of *E*-fluvoxamine (2-[(*E*)-[5-methoxy-1-[4-(trifluoromethyl)phenyl]pentylidene]amino]oxyethanamine) to its *Z*-isomer [22]. Thus, given that *Z*-GIZH-298 exists as a defined geometric isomer of a substituted oxime, a thorough assessment of its propensity for *E/Z*-isomerization is required. Such analysis is essential for understanding the structural stability of the compound and its behavior under various experimental and storage conditions.

The synthetic route to GIZH-298 involves the initial formation of both *E*- and *Z*-isomers of 4-benzoylpyridine oxime [15]. While recrystallization effectively isolates the *Z*-isomer, trace amounts of the *E*-isomer may still remain through subsequent synthetic steps. Therefore, the comprehensive characterization of both isomeric forms—including their physicochemical properties and their propensity for interconversion—is essential. Such characterization is critical for pharmaceutical development, as it ensures accurate assessment of compound purity and supports the long-term stability of *Z*-GIZH-298 as a drug candidate.

## 2. Results and Discussion

### 2.1. Synthesis of GIZH-298 Isomers

**Scheme 2:**
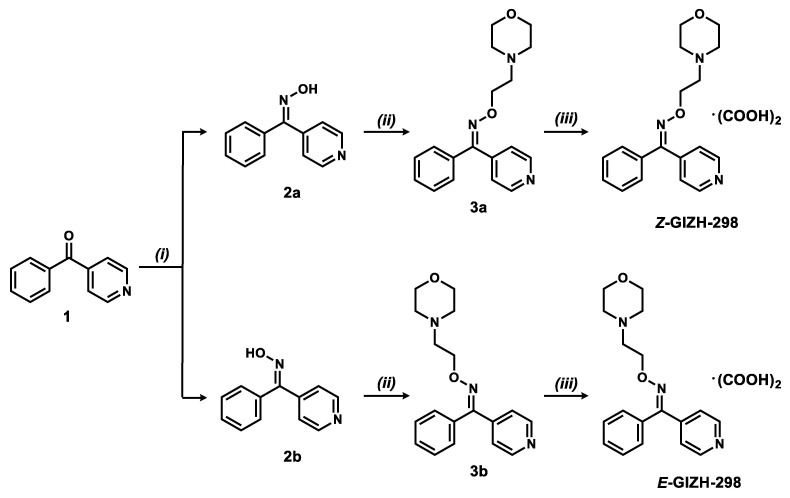
Synthesis of GIZH-298 isomers. Reagents and conditions: (i) NH_2_OH*HCl, NaOH, EtOH, water, reflux, 4 h; (ii) 4-(2-chloroethyl)morpholine, NaH, DMF, rt, 24 h; (iii) oxalic acid, EtOH.

*Z*-GIZH-298 is the oxalate salt of the *Z*-oxime of 4-benzoylpyridine substituted with the morpholinoethyl moiety. At the Medicinal Chemistry Department of the Federal Research Center for Innovators and Emerging Biomedical and Pharmaceutical Technologies, *Z*-GIZH-298 was synthesized from commercially available 4-benzoylpyridine (**1**) through a three-step sequence: condensation of 4-benzoylpyridine (**1**) with hydroxylamine under basic conditions to afford oxime (**2a**); alkylation of oxime (**2a**) with 2-chloroethylmorpholine to give the substituted oxime (**3a**); and finally, conversion of oxime (**3a**) into the oxalate salt [15] (Scheme 2).

**Figure 2:**
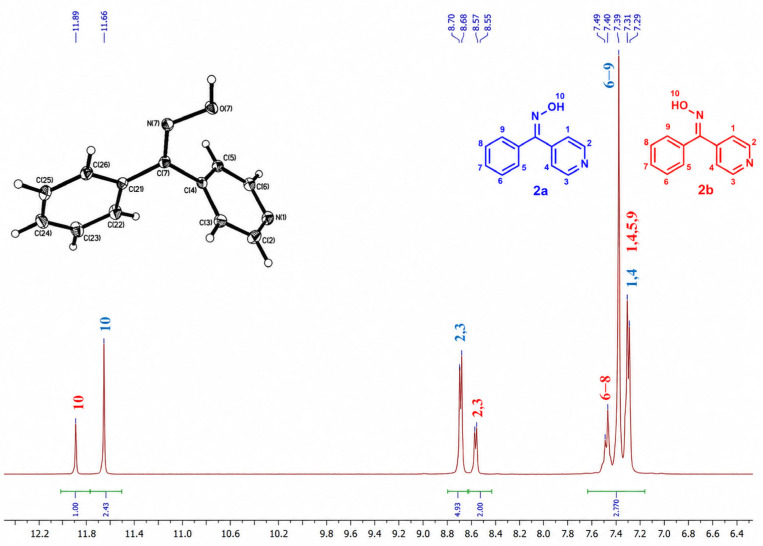
Preparation of a mixture of 4-benzoylpyridine oxime isomers and their ratio based on NMR data. Red—*E*-form, blue—*Z*-form. Literature X-ray of 4-benzoylpyridine Z-oxime [23].

In the first step, a mixture of the *E*- and *Z*-isomers of 4-benzoylpyridine oxime **2a** and **2b** is formed (Figure 2) in a ratio of *E:Z* = 1:2.5, as determined by ^1^H NMR spectroscopy.

The (*Z*)-phenyl(pyridin-4-yl)methanone oxime (**2a**) exhibits lower solubility in ethanol and can be isolated by recrystallization from ethanol in 47% yield. The melting point and ^1^H and ^13^C NMR spectrum of the *Z*-isomer are consistent with the previously published data [24]. For crystals of the *Z*-isomer obtained under similar conditions, the literature provides X-ray diffraction data confirming the molecular structure [23] (Figure 2).

The mother liquor remaining after filtration of the crude *Z*-isomer consisted of a mixture of the *E*- and *Z*-oximes in a ratio of 2.5:1. According to literature data [25], *E*-oxime isomers exhibit lower solubility in chloroform, which made it possible—through three successive recrystallizations of the concentrated mother liquor from this solvent—to isolate the *E*-isomer of 4-benzoylpyridine oxime **2b** in a yield of 10%.

Thus, due to the differences in solubility, the *E*- and *Z*-isomers of 4-benzoylpyridine oxime were finally obtained in the pure forms.

At the next stage, the alkylation reactions of the isolated oximes with 2-chloroethylmorpholine were carried out, yielding the target compounds *Z*-GIZH-298 and *E*-GIZH-298 as their oxalate salts (Scheme 2). The physicochemical, spectral, and biological distinctions between these isomers are described below.

### 2.2. Comparison of Spectral and Physicochemical Characteristics of the GIZH-298 Isomers

#### 2.2.1. NMR Spectra

**Figure 3:**
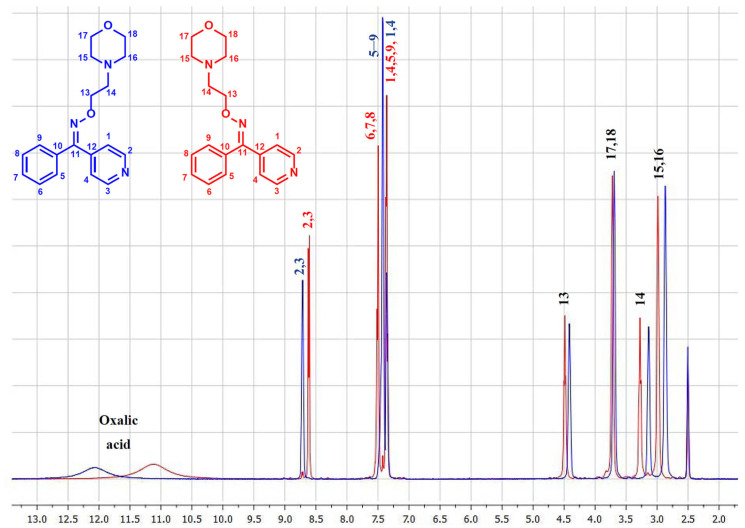
Comparison of ^1^H NMR spectra of GIZH-298 geometric isomers. Red—*E*-form, blue—*Z*-form.

As noted above, the *E*- and *Z*-isomers of GIZH-298 exhibit distinct features in their NMR spectra. Figure 3 shows the ^1^H NMR spectra of the two isomers (red: *E*-form; blue: *Z*-form). Notably, small but reproducible differences are observed in the chemical shifts in the protons at positions 2 and 6 of the pyridine ring (Δδ ≈ 0.15 ppm), as well as in the aromatic protons of the phenyl ring (Δδ ≈ 0.1 ppm in both the downfield and upfield regions). These variations are likely attributable to differences in the spatial arrangement of the oxime functional group, including the shielding effect of the oxime oxygen lone-pair electrons, geometric factors, and steric interactions between the morpholinoethyl moiety and the aromatic rings.

**Figure 4:**
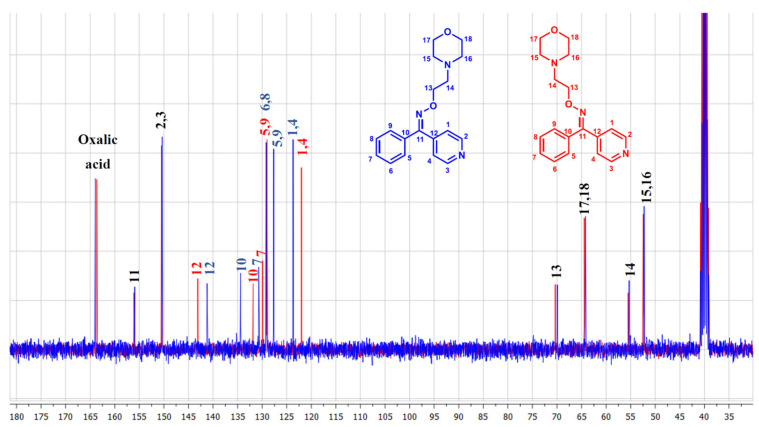
^13^C NMR spectra for geometric isomers of GIZH-298. Red—*E*-form, blue—*Z*-form.

Slight differences in the chemical shifts of several carbon atoms in the geometric isomers of GIZH-298 are also evident in the ^13^C NMR spectra. In particular, the C-2 and C-6 atoms of the phenyl ring in the *E*-isomer appear slightly downfield (128.9 ppm) relative to the corresponding signals in the *Z*-isomer (127.6 ppm) (Figure 4). The inverse relationship is observed for C-2 and C-6 of the pyridyl ring. In the *E*-isomer, both carbons appear at δ 122.0 ppm, whereas in the *Z*-isomer these signals are shifted downfield to δ 123.7 ppm. Differences are also observed for the tertiary carbons of the phenyl ring: in the *E*-isomer, this signal appears at 131.8 ppm, whereas in the *Z*-isomer it is shifted downfield to 134.4 ppm. A similar and inverse difference is observed for the quaternary pyridine carbon atoms: 141.2 ppm for the *Z*-isomer and 143.1 ppm for the *E*-isomer.

#### 2.2.2. IR Spectra

**Figure 5:**
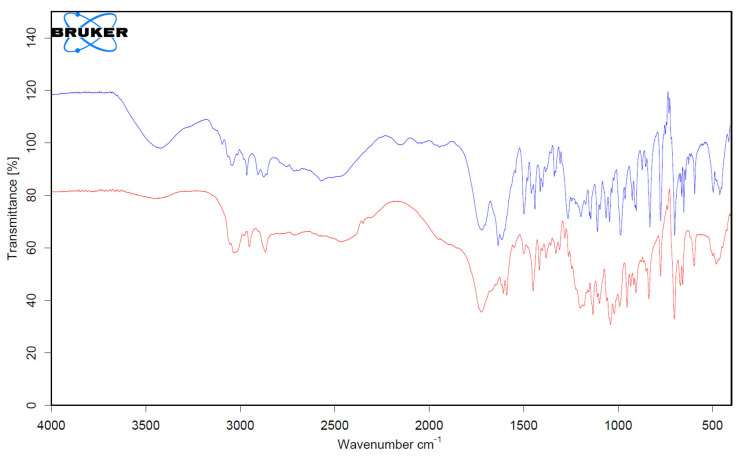
IR spectra of *Z*- and *E*-isomers of GIZH-298. Red—*E*-form, blue—*Z*-form.

The IR spectra of the two isomers are shown in Figure 5. The IR spectrum of *Z*-GIZH-298 exhibited the following absorption bands (cm^−1^): 3424—–O-H stretching vibrations of the oxalate, 3041—C-H stretching vibrations of aromatic CH groups, 2965, 2906, 2874—C-H stretching vibrations of aliphatic CH_2_ groups, 2400–2600 (range)—a broad band of N^+^H stretching vibrations, 1721—C=O stretching vibrations of oxalic acid appeared as a broad band, 1400–1700 (range)—overlapping bands of C=N stretching, C=O stretching of the oxalate anion, and aromatic C=C stretching vibrations (1635, 1614, 1498, 1459), 1265 and 1144—C-O stretching vibrations of the carboxyl group of the oxalate and the morpholine ring, 987—N-O vibrations.

The IR spectrum of *E*-izomer exhibited the following absorption bands (cm^−1^): 3452—O-H stretching vibrations of the oxalate, 3055, 3035—C-H stretching vibrations of aromatic CH groups, 2952, 2867—C-H stretching vibrations of aliphatic CH_2_ groups, 2400–2600 (range)—a broad band of N^+^H stretching vibrations, 1723—C=O stretching vibrations of oxalic acid appeared as a broad band, overlapping with the C=N and oxalate anion C=O stretching band (around 1640–1660), 1400–1600 (range)—aromatic C=C stretching vibrations (1606, 1589, 1496, 1450), 1230–1180 (range)—C-O stretching vibrations of the carboxyl group of the oxalate and the morpholine ring appeared as a broad, overlapping band, 990—N-O vibrations.

As can be seen in Figure 5 and from the description above, certain differences are observed in the spectra of the *Z*- and *E*-isomers. However, a clear determination of the position and intensity of the stretching vibration band of the C=N bond of the oxime group, which could provide information about the indicated structural differences, is complicated by the presence of strong absorption bands from the C=O bonds of both ionized and non-ionized carboxyl groups of the oxalic acid.

#### 2.2.3. UV Spectra

**Figure 6:**
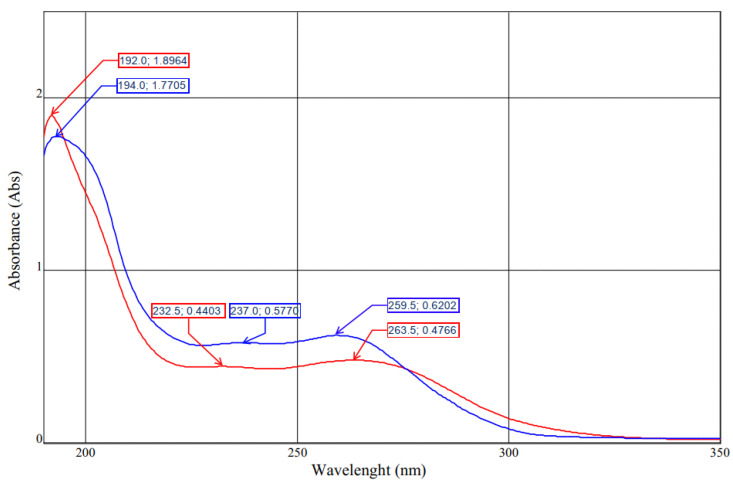
UV absorbance plots of *Z*- and *E*-isomers of GIZH-298. Red—*E*-form, blue—*Z*-form.

UV-*Vis* spectra of the *Z*- and *E*-isomers of GIZH-298 were recorded in the 190–350 nm wavelength range at a concentration of 0.02 mg/mL in the purified water. The resulting spectra are presented in Figure 6. The absorption spectra of both geometric isomers of GIZH-298 exhibit two weakly expressed maxima in the regions of 230–240 nm and 250–270 nm, which are presumably attributed to π-π* and n-π* electronic transitions within the conjugated system. The slight multidirectional shifts in the absorption maxima positions of the isomers (≤5 nm) in the above-mentioned regions preclude a definitive assessment of the influence of geometric isomerism on the spectral profile. Also, the spectrum of *Z*-GIZH-298 shows a hyperchromic effect relative to that of the *E*-isomer, which is likely due to its more flexible π-electron system.

#### 2.2.4. Melting Points

The melting points determined for the intermediate 4-benzoylpyridine oxime isomers were 175–180 °C for the *Z*-isomer and 158–162 °C for the *E*-isomer. Differences in the melting points between geometric isomers are well documented in the literature [26,27]. In many cases, *E*-isomers exhibit lower melting points than their corresponding *Z*-isomers, a trend generally attributed to differences in molecular geometry and the resulting hydrogen-bonding patterns within the crystal lattice [27,28]. For the final compounds, *E*-GIZH-298 and *Z*-GIZH-298, the melting points differ to a lesser extent: 158–161 °C for the *Z*-form and 165–168 °C for the *E*-form.

#### 2.2.5. HPLC and Mass Spectrometry

**Figure 7:**
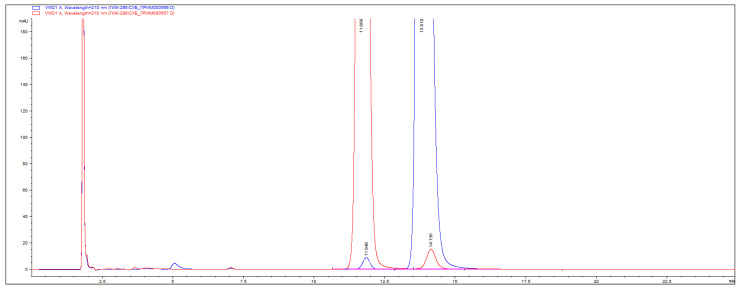
Representative overlay of chromatograms of the test solution of the substance *E*-GIZH-298 at a concentration of 1 mg/mL (red) and the test solution of the substance *Z*-GIZH-298 at a concentration of 1 mg/mL (blue).

As part of the standard studies performed to develop an HPLC analytical procedure for identifying related impurities in the *Z*-GIZH-298 active pharmaceutical ingredient, one of the detected impurities was found to exhibit a retention time consistent with that of the *E*-isomer. Figure 7 presents an overlay of the chromatograms of *Z*-GIZH-298 and *E*-GIZH-298 substances. The retention time of *Z*-GIZH-298 was about 14 min, *E*-GIZH-298—about 12 min. The relative retention time of the *E*-isomer was 0.86.

Chromatographic purity of *Z*-GIZH-298 and *E*-GIZH-298 substances calculated by internal normalization method was 99.32% and 98.84% respectively (Figure 7).

The presence of *E*-GIZH-298 as a related substance in the compound *Z*-GIZH-298 requires additional confirmation of its structure by mass-spectrometric analysis. For this purpose, other chromatography conditions were selected, which made it possible to resolve *Z*-GIZH-298 and its impurity and to use the eluate for mass spectrometric analysis.

**Figure 8:**
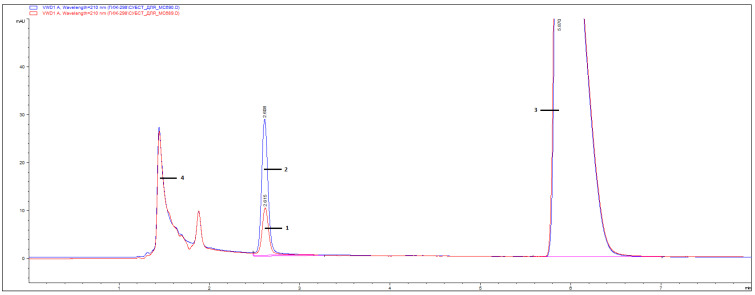
Representative chromatogram overlay of the *Z*-GIZH-298 test solution (batch 260324, concentration 0.05 mg/mL) without (red) and with the *E*-isomer addition (blue) (1—the impurity peak observed in the substance; 2—the impurity peak observed in the substance spiked with the *E*-isomer; 3—*Z*-GIZH-298 main peak; 4—oxalic acid peak).

*Z*-GIZH-298 test solution (batch 260324) with a significant content of the studied impurity (1.45%) was analyzed, then 300 µL of an *E*-isomer solution with a concentration of 0.05 mg/mL was added to the above solution (for identification by the spike test). The obtained chromatogram overlay is shown in Figure 8. As it can be seen from Figure 8, the retention time of the studied impurity also corresponds to the *E*-isomer GIZH-298.

The fraction of the studied impurity was collected within the chromatographic analysis of the *Z*-GIZH-298 test solution (estimated impurity content is about 1.5 micrograms in 8 mL of eluate).

The following samples were analyzed using a mass spectrometric detector:

- *Z*-GIZH-298 substance (batch 260324, *E*-isomer impurity content is 1.45% according to HPLC analysis);
- *E*-GIZH-298 (*Z*-GIZH-298 impurity content is 7.6%);
- studied impurity fraction collected within a 5-fold chromatographic run on an LC column.

**Figure 9:**
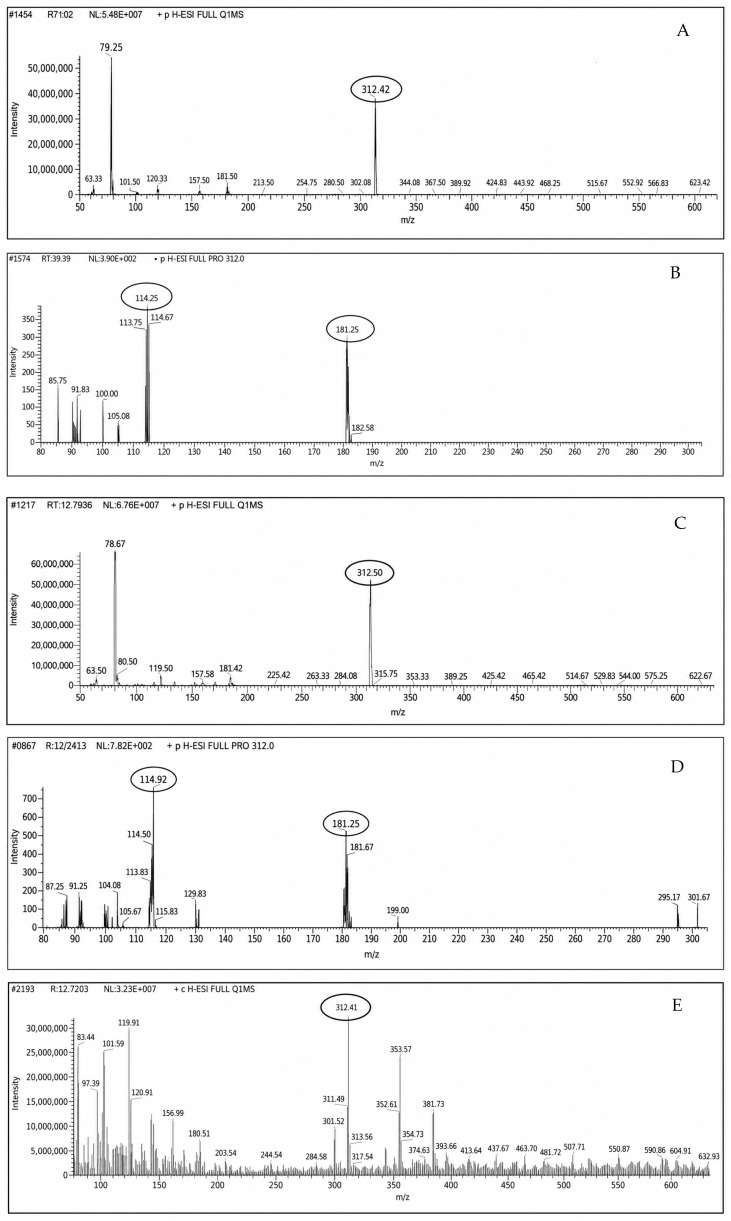

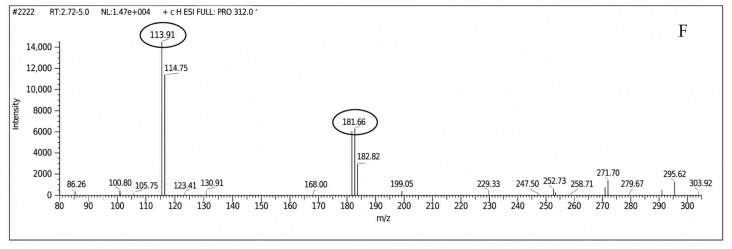
Mass spectra of *Z*-GIZH-298 (**A**,**B**), *E*-GIZH-298 (**C**,**D**) and collected HPLC fraction of the studied impurity (**E**,**F**): (**A**,**C**,**E**) Q1 mode, Total Ion Current; (**B**,**D**,**F**) Product Ions mode.

The resulting mass spectra for the test samples are shown in Figure 9. The results of studying the electrospray ionization of target compounds are presented in Table 3.

Thus, for both the *Z*-isomer of GIZH-298 and the *E*-isomer, as well as for the fraction of the main impurity on HPLC, corresponding in retention time to the *E*-isomer, the same precursor ion (molecular ion) was detected in the mass spectra—312 *m*/*z* and also specific products of its decomposition—114 and 181 *m*/*z*. This confirms the correspondence of the impurity present in the samples of *Z*-GIZH-298 substance to its *E*-isomer.

### 2.3. X-Ray Diffraction Analysis (XRD)

**Figure 10:**
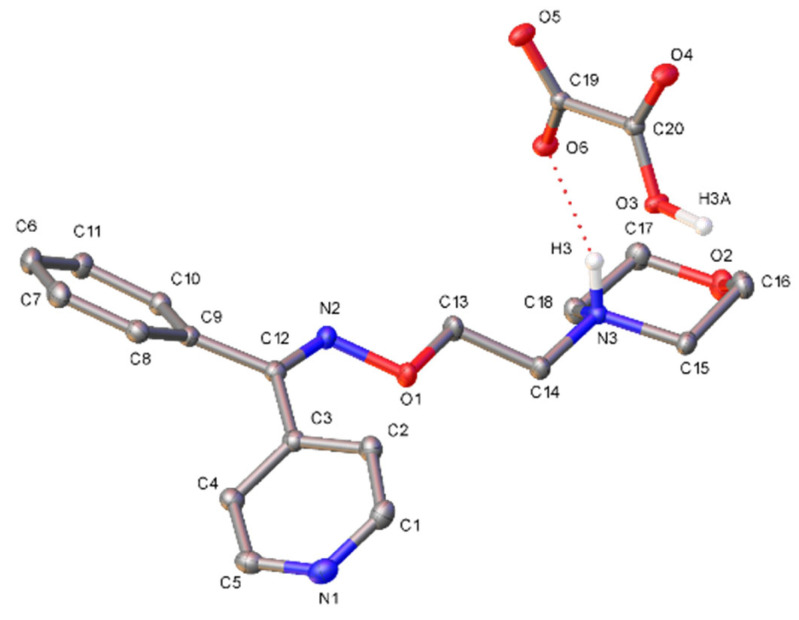
General view of *Z*-GIZH-298. Thermal ellipsoids of atomic displacement are presented at 70% probability. The color coding of the atoms is as follows: red—oxygen, blue—nitrogen, gray—carbon, and white—hydrogen. Hydrogen atoms not involved in intramolecular interactions are omitted for clarity. The dashed line indicates a hydrogen bond with a significant structural influence.

To absolutely confirm the molecular configuration of *Z*-GIZaccH-298, single-crystal X-ray diffraction analysis was performed (Figure 10). Hydrogen atoms bound to carbon were placed in the calculated positions and refined using a riding model, while O–H hydrogen atoms were located from the electron-density difference map and refined freely. The key crystallographic and refinement parameters are summarized in Supplementary Materials.

**Figure 11:**
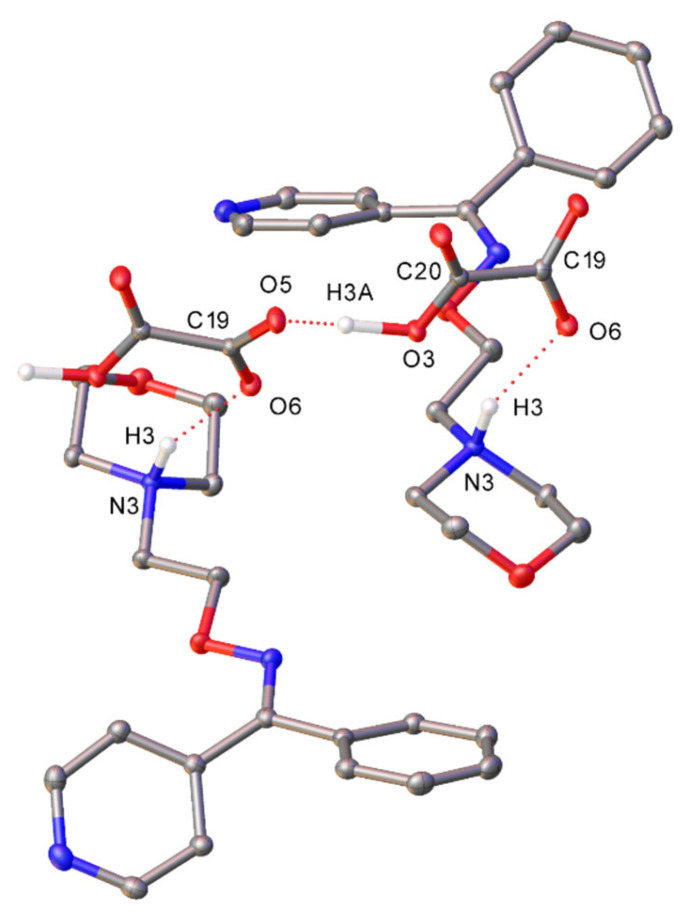
Visual representation of coordinated covalent bond N3-H3…O6. The color coding of the atoms is as follows: red—oxygen, blue—nitrogen, gray—carbon, and white—hydrogen. Hydrogen atoms not involved in intramolecular interactions are omitted for clarity. The dashed line indicates a hydrogen bond with a significant structural influence.

According to the crystal structure of *Z*-GIZH-298, it crystallizes in the P2_1_/c space group. The asymmetric unit of the cell comprises one molecule of the compound (Figure 11).

Notably, one of the hydrogen atoms from oxalic acid has a protonated N3 atom with an N3-H3...O6 interaction; hence, oxalic acid remains deprotonated. Therefore, analysis of the crystal package revealed 3 distinct intermolecular hydrogen bonds: two N3-H3…O6 and one O5-H3A…O3, hence molecules pack into 1D polymeric chains. The angles and distances of all intermolecular hydrogen bonds are listed in Supplementary Materials. N2-O1 distance is 1.41 Å, which indicates a single bond, whereas C12-N2 bond distance is 1.29 Å, which indicates both a N-C double bond and a strong electron-withdrawing system of the phenyl and pyridyl groups. No π-π stacking interactions have been noted.

### 2.4. Study of the Influence of Various Conditions on the Isomerization of the Z-Isomer GIZH 298 into the E-Isomer

#### 2.4.1. Study of Photostability of Z-GIZH-298

Within the photostability test, the *Z*-GIZH-298 sample was exposed to light at a total light exposure of 1.2 million lux-h and a near-ultraviolet energy exposure of 480 W × h/m^2^ [29]. The control sample was stored in a place protected from light at room temperature. According to the results of HPLC analysis for related impurities, the *E*-isomer GIZH-298 concentration in the test sample increased significantly compared to the control sample. The *E*-isomer GIZH-298 content changed from 0.41% to 9.75% (relative retention time 0.86). The results obtained indicate that light radiation initiates the transition from the *Z*-isomer of GIZH-298 to the *E*-isomer.

#### 2.4.2. Study of the Thermostability of Z-GIZH-298

The experiments within the study evaluated the *Z → E* configurational conversion of 4-benzoylpyridine oxime (**2a**) in solution. Toluene (bp 110 °C), a high-boiling nonpolar solvent, and dioxane (bp 101 °C), a polar aprotic solvent, served as model media. Heating in toluene resulted in no detectable isomerization (*Z:E* = 100:0), whereas dioxane promoted a slight enrichment of the *E*-isomer (*Z:E* = 94:6).

The experiments assessing the configurational stability of *Z*-GIZH-298 as a function of temperature and solvent polarity were also conducted (Table 4). All solutions were handled under light-excluded conditions. Water (polar protic), dioxane (polar aprotic), and toluene (nonpolar) were employed as representative solvents. Samples containing 0.5 g of *Z*-GIZH-298 were heated under reflux for 5–24 h, followed by analysis of the resulting mixtures by ^1^H NMR spectroscopy. Heating in the nonpolar medium produced no detectable configurational change, consistent with the calculated energy barrier. Dioxane generated approximately 4% of the *E*-isomer, whereas heating in water for 5 h yielded 5% of the *E*-isomer. Prolonged heating in water (24 h) increased the *E*-isomer content to 9% (Table 4).

#### 2.4.3. Studying the Influence of the pH

**Figure 12:**
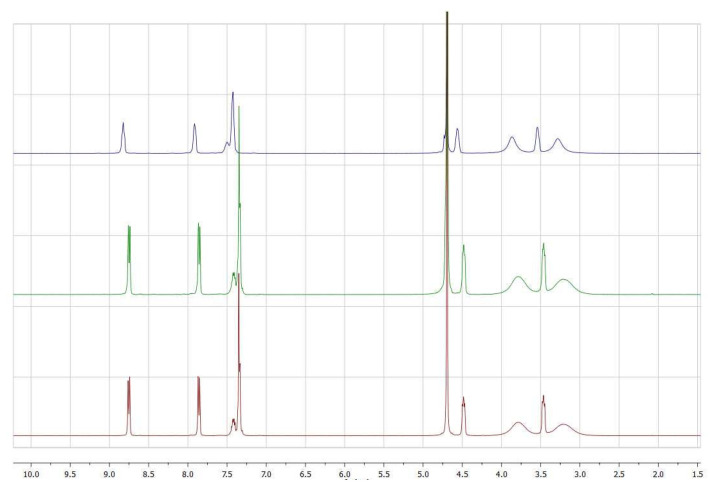
Analysis of *Z*-GIZH-298 stability in D_2_O water (blue—0 h, green—2 days, red—8 days).

**Figure 13:**
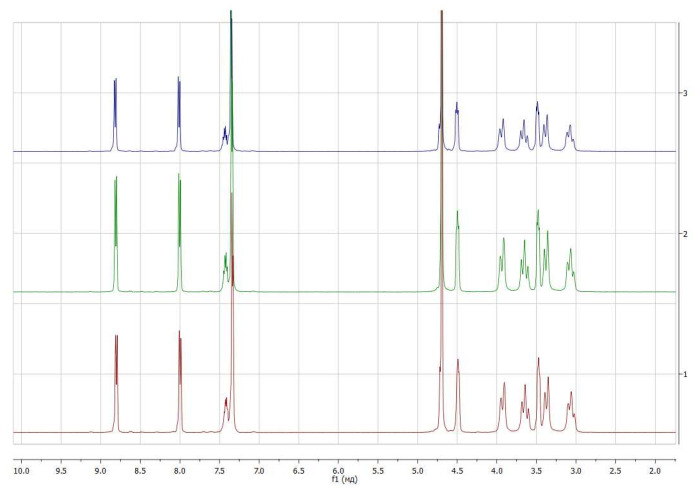
Analysis of the stability of *Z*-GIZH-298 in trifluoroacetic acid (TFA-*d4*) (blue—0 h, green—2 days, red—8 days).

The stability of *Z*-GIZH-298 in aqueous and acidic media at ambient temperature was also examined. Samples of *Z*-GIZH-298 were maintained in D_2_O and in D_2_O containing deutero-trifluoroacetic acid for an 8-day period. Figure 12 and Figure 13 summarize the resulting data. No appreciable changes appeared in either sample over the course of the experiment, indicating that the *Z*-isomer of the substituted oxime retains its configuration under these conditions.

#### 2.4.4. Calculations of the Energy Barrier for the Transition of Isomers

**Figure 14:**
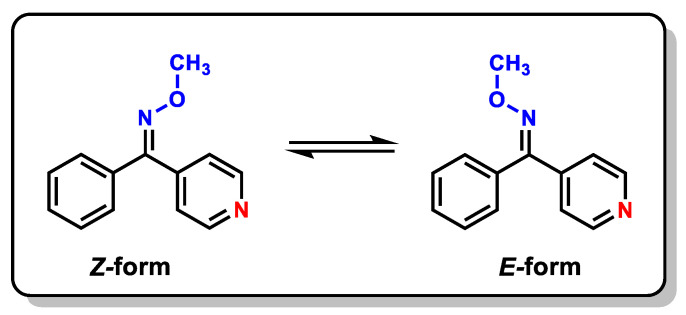
Compound structures proposed for transition energy calculations.

As a part of the study, the ab initio calculations were carried out in order to elucidate the mechanism of photoisomerization of the potential antiepileptic agent *Z*-GIZH-298. The ethylmorpholine substituent of *Z*-GIZH-298 is not conjugated with the main π-system and therefore does not contribute to electronic transitions, so in computations it was replaced with a methyl group (Figure 14).

Final electronic energies for all stationary points and scan geometries, both in the ground (S_0_) and first excited (S_1_) states, were obtained at the multistate-CASPT2/ANO-RCC-VDZP level. The π- and π*-type orbitals were used as the active space for the state-averaged complete active space self-consistent field (SA-CASSCF) reference, corresponding to (14e, 14o) for the model molecule. The three lowest states, identified from preliminary TD-DFT calculations at the BHandHLYP/def2-TZVPD level, were included in state averaging. According to Merritt et al. [30], a further increase of the basis set in CASPT2 calculations does not improve the result in the photoisomerization reaction of azobenzene.

**Ground-state pathway.** Initial geometries for the *Z*- and *E*-isomers were generated at the MP2/def2-TZVP/PCM (toluene) level. These structures were then used as starting points for relaxed potential energy surface (PES) scans along the Py-C=N-OMe dihedral angle, performed at the SA-CASSCF(14,14)/ANO-RCC-VDZP/PCM(toluene) level, with all remaining internal coordinates re-optimized at each scan point. The resulting CASSCF-optimized geometries of *Z*, *E*, and TS (*S*_0_) were used for final energy refinement at the multistate-CASPT2/ANO-RCC-VDZP/PCM(toluene) level via single-point calculations.

**Excited-state pathway**. Geometry optimization along the S_1_ surface was performed at the TD-BHandHLYP/def2-TZVPD level, since stable CASSCF/CASPT2 optimization of the S_1_ surface for this system was not feasible. The use of TD-DFT with high exact-exchange hybrid functionals for excited-state geometry optimization, followed by multireference single-point energy refinement, is supported by benchmark studies on comparable π → π* photoisomerization systems [31,32].

All calculations were carried out using the Conductor-like Polarizable Continuum Model (CPCM). Since the behavior of the GIZH-298 molecule was modeled in a crystalline-like environment, where it is surrounded by similar molecules, the dielectric constant of the medium was set to that of toluene, which has a polarizability comparable to GIZH-298 owing to the presence of multiple aromatic fragments. All multireference calculations were performed using the OpenMolcas v.24.02; DFT and MP2 calculations were carried out in ORCA 5.0.4.

Multistate-CASPT2 calculations on the optimized *S*_0_ geometries reproduce the absorption spectra and key bond lengths of both isomers obtained from X-ray diffraction analysis (Table 5), supporting the adequacy of the chosen level of theory for describing the S_1_ excited state as well. Based on the calculated data, the *E*-isomer is more stable than the *Z*-isomer by ~2 kcal/mol.

**Figure 15:**
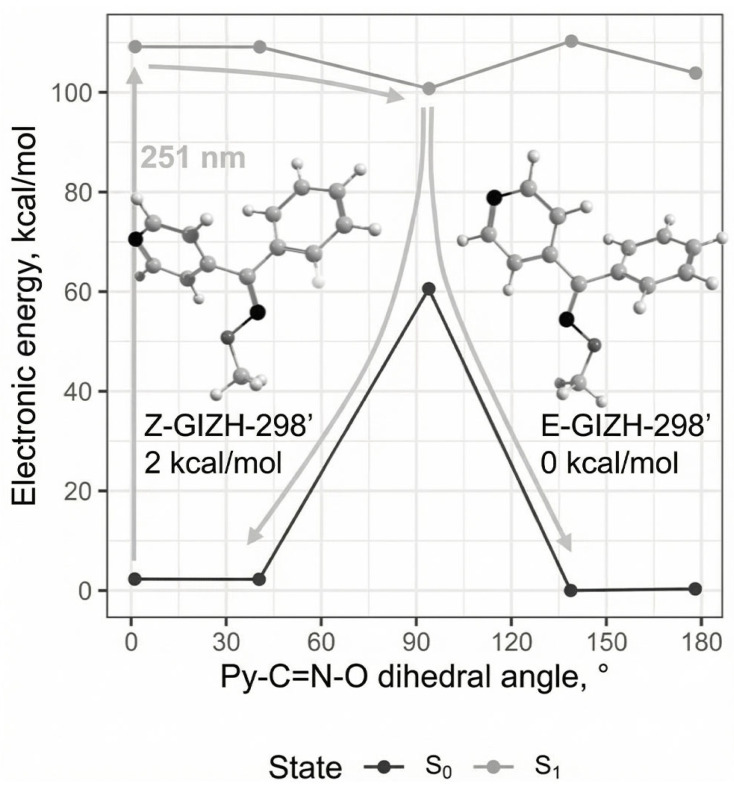
PES slice along the rotation of the Py-C=N-OMe dihedral angle in the model molecule in the ground (*S*_0_) and first singlet excited (*S*_1_) states. The grey arrows show the excitation-initiated isomerization pathway.

In order to directly study the process of photoisomerization, the potential energy curves along the rotation of the Py-C=N-OMe dihedral angle in the *S*_0_ and *S*_1_ states were calculated (Figure 15).

The activation energy of isomerization through the ground state is about 60 kcal/mol, which makes thermal isomerization of the unexcited molecule effectively impossible at temperatures below 600 °C. In contrast, the S_1_ isomerization barrier is small, amounting to only ~10 kcal/mol. Following S_0_ → S_1_ excitation, the system relaxes towards a twisted conformer with a CCNO dihedral of approximately 90°. Subsequent S_1_ → S_0_ relaxation from this conformer can proceed either back to the Z-form or forward to the E-form, leading to a gradual photochemical accumulation of the *E*-isomer.

The thermal *Z → E* conversion of GIZH-298 observed upon prolonged heating in polar solvents (Section 2.4.2), although limited in extent, indicates that some pathways available under these conditions are not captured by the present continuum description contribute to the isomerization. A detailed elucidation of these solvent-mediated channels lies beyond the scope of the present study.

### 2.5. Comparison of Anticonvulsant Pharmacological Properties of GIZH-298 Isomers

As was noted in the introduction, *Z*-GIZH-298 exhibits a broad anticonvulsant activity profile across a wide range of animal models. Since the *E*-isomer of the compound is present as an impurity in the main substance and can also be accumulated during storage, it was important to assess the intrinsic activity of this isomer. To compare the anticonvulsant properties of the *E*- and *Z*-isomers of GIZH-298, blinded experiments were carried out using the MES test in mice (Table 6). *Z*-GIZH-298 at 100 mg/kg peroral (p.o.) exhibited pronounced anticonvulsant efficacy, providing 100% protection against the tonic hind limb extension and reducing the median seizure severity score to 2 (vs. 4 in control). When the dose was reduced to 60 mg/kg, the effect diminished yet remained statistically significant relative to control values for the tonic phase. The *E*-isomer of GIZH-298 at 100 mg/kg (p.o.) produced a moderate but statistically significant reduction in the proportion of animals exhibiting tonic hind limb extension (27% vs. 91% in controls). The median seizure severity score was also reduced to 2; however, the response was heterogeneous, ranging from 2 to 4 points. Reducing the dose of *E*-GIZH-298 to 60 mg/kg abolished the anticonvulsant effect entirely. Thus, both geometric isomers of GIZH-298 display anticonvulsant activity, but with markedly different potencies: the *Z*-isomer is substantially more effective than the *E*-isomer. In terms of overall efficacy, *Z*-GIZH-298 is comparable to sodium valproate and even exceeds it in potency.

These results further confirm the robust anticonvulsant properties of the *Z*-isomer of GIZH-298, demonstrating that it is at least comparable, if not superior, in efficacy to the current gold standard for epilepsy therapy, sodium valproate. The *Z*-isomer consistently provided complete protection against tonic hind limb extension and significantly reduced seizure severity even at lower doses, highlighting its strong therapeutic potential. In contrast, while the *E*-isomer exhibits some anticonvulsant activity, its effect is less pronounced and is highly dose-dependent, underscoring the critical role of the *Z*-configuration in mediating the pharmacological response. Overall, these findings provide compelling evidence for prioritizing *Z*-GIZH-298 in further preclinical development as a potent and reliable antiepileptic agent.

### 2.6. An Assessment of Genotoxicity of GIZH-298 Isomers

Antiepileptic drugs as a class are known to exhibit mutagenic or genotoxic properties in various experimental systems. Consequently, international regulatory guidelines, including ICH S2(R1) and ICH M7, require rigorous assessment to ensure that the new drug candidates do not pose DNA-reactive or genome-damaging risks. For novel anticonvulsant agents such as *Z*-GIZH-298, the absence of mutagenic and genotoxic effects is therefore a critical prerequisite for further development. This requirement extends not only to the parent compound but also to all potential impurities and photochemically generated products, including *E/Z*-isomers, which must likewise be evaluated in accordance with photo safety guidance (ICH S10). Ensuring that both the drug and any possible photoproducts are devoid of genotoxic properties is essential for establishing their safety profile and suitability for clinical progression.

*Z*-GIZH-298 underwent mutagenicity screening at concentrations of 1.6, 8, 40, 200, 1000, and 5000 µg/mL in *Salmonella typhimurium* strains TA98, TA100, TA1535, and TA1537, as well as in the combined *Escherichia coli* strains pKM101 and uvrA. Evaluations proceeded both with and without metabolic activation (S9 fraction), and none of the tested concentrations produced mutagenic responses [33,34,35,36,37,38,39,40].

Table 7 summarizes the cytogenetic profile of *Z*-GIZH-298. Single intraperitoneal administration at 40, 100, and 200 mg/kg to male mice, along with repeated intraperitoneal dosing at 40 mg/kg to both sexes, yielded no chromosomal aberrations in bone marrow cells.

Data in Table 8 describe the DNA-damage assessment performed in vivo in multiple mouse tissues under 3 and 24 h exposure conditions. *Z*-GIZH-298, administered intraperitoneally at 40 and 200 mg/kg, did not elicit detectable DNA damage in bone marrow, liver, kidney, or spleen cells at any time point. The collective results from short-term mutagenicity assays and DNA integrity evaluations indicate that *Z*-GIZH-298 does not exhibit characteristics consistent with carcinogenic potential.

Based on these findings, the *E*-GIZH-298 underwent testing only in the Ames assay using the minimal required set of Salmonella typhimurium strains (TA98, TA100, and TA1537). At the tested concentrations of 0.4, 2, 10, 50, 250, and 1250 µg/mL, the *E*-isomer produced no mutagenic effects in the presence or absence of metabolic activation (S9 fraction).

Taken together, these results support the genotoxic and potential carcinogenic safety profile of *Z*-GIZH-298.

## 3. Materials and Methods

The melting points of the compounds were determined using the Optimelt MPA100 instrument (Stanford Research Systems, Sunnyvale, CA, USA) in the open melting-point tubes without correction. The structures of the target and intermediate compounds were determined by one-dimensional ^1^H-, ^13^C- and two-dimensional (COSY—homonuclear correlation, HSQC—heteronuclear single quantum correlation) NMR spectroscopy. ^1^H- and ^13^C-NMR spectra were recorded on the δ scale (ppm) on a Fourier 300 HD spectrometer (Bruker Corporation, Bremen, Germany; 300 and 75 MHz for ^1^H- and ^13^C nuclei, respectively) in DMSO-*d6* solutions with tetramethylsilane (0 ppm) and signals of the residual solvent protons observed at δ 2.50 for DMSO-*d6* as internal standard. The spin–spin interaction constant is *J*, Hz. The following abbreviations were used to designate resonant signals: s—singlet, d—doublet, t—triplet, q—quartet, and m—multiplet. TLC was performed on aluminum-backed silica gel plates (Merck DC, Alufolien Kieselgel 60 F254) with spots visualized by UV light. All solvents were reagent grade, and when necessary, were purified and dried by standard methods. The concentration of solutions after reactions and extractions involved the use of a rotary evaporator operating at a reduced pressure of 20 Torr. Organic solutions were dried over anhydrous sodium sulfate.

IR spectra were recorded on a Bruker INVENIO X FT-IR spectrometer, software—OPUS Version 8.5 (SP1) (Bruker Optik GmbH, Ettlingen, Germany), in KBr discs (2 mg of substance per 200 mg of KBr). The following abbreviation was used to designate absorption bands: br—broad.

UV spectra were obtained on a Unico 2804 spectrophotometer (United Products and Instruments, USA); measurements were carried out in Hellma Analytics (QS) quartz cuvettes with a layer thickness of 10 mm (Hellma GmbH & Co. KG, Müllheim, Germany).

### 3.1. Synthesis

#### 3.1.1. Synthesis of 4-Benzoylpyridine Oximes

**(*Z*)-phenyl(pyridin-4-yl)methanone oxime (2a).** 4-Benzoylpyridine (25.00 g, 0.13 mol) was dissolved with stirring on a magnetic stirrer in 135 mL of ethyl alcohol at room temperature and the solution of hydroxylamine hydrochloride (28.49 g, 0.41 mol) in 30 mL of water was added dropwise during 5 min. A solution of sodium hydroxide (13.67 g, 0.34 mol) in 20 mL of water was then added dropwise during 15 min. The reaction mixture was then refluxed with stirring for 4 h. Then heating was discontinued, and the reaction mixture was cooled to room temperature and left overnight without stirring. The precipitate was filtered off and washed with water (3 × 200 mL) and ethanol (50 mL). It was then dried in air, yielding 19.65 g of a white solid which consisted of 20% of *E*-oxime and 80% of *Z*-oxime, according to ^1^H NMR. It was then dissolved in 230 mL of boiling ethanol; the hot solution was filtered and left to crystallize overnight. The crystals were filtered, washed with ethanol (2 × 25 mL) and air-dried to afford 12.90 g (47%) of the desired oxime as a white solid. MP. = 178–183 °C. R*_f_* = 0.50 (CH_2_Cl_2_:methanol = 10:1). ^1^H NMR (DMSO-*d6*, δ, ppm., *J*/Hz): 7.30 (d, 2H, *J* = 5.9 Hz, 3,5-H_Py_), 7.34–7.41 (m., 5H, H_Ph_), 8.68 (d, 2H, *J* = 5.9 Hz, 2,6-H_Py_), 11.68 (s, 1H, NOH).

^13^C NMR (DMSO-*d6*, δ, ppm.): ^13^C NMR (DMSO-*d6*, δ, ppm.): 124.1 (2*C, 3,5-C_Py_), 127.2 (2*C, 2,6-C_Ph_), 129.0 (2*C, 3,5-C_Ph_), 129.7 (4-C_Ph_), 135.7 (C_Ph_), 141.8 (C_Py_), 150.2 (2,6-C_Py_), 153.8 (C=N).

The aqueous-ethanol filtrate was saved for the isolation of the *E*-oxime (see below).

**(*E*)-phenyl(pyridin-4-yl)methanone oxime (2b).** The mother liquor from the synthesis of (*Z*)-phenyl(pyridin-4-yl)methanone oxime (see above) was filtered, and the solid on the filter was washed with water (5 × 100 mL) and dried in the air, affording a white powder which consisted of 73% of *E*-oxime and 27% of *Z*-oxime according to ^1^H NMR. To isolate the *E*-isomer, a portion of the dry residue of the mother liquor weighing 2.5 g was taken. It was then recrystallized 3 times from chloroform (50 mL, 20 mL, 15 mL), yielding 0.5 g (10% calculated per portion) of the *E*-oxime as a white solid. MP. = 158–162 °C. R*_f_* = 0.50 (CH_2_Cl_2_:methanol = 10:1). ^1^H NMR (DMSO-*d6*, δ, ppm., *J*/Hz): 7.27–7.39 (m., 4H, 2,6-H_Ph_, 3,5-H_Py_), 7.43–7.55 (m, 3H, 3,4,5-H_Ph_),8.57 (d, 2H, *J* = 4.7 Hz, 2,6-H_Py_), 11.87 (s, 1H, NOH).

^13^C NMR (DMSO-*d6*, δ, ppm.): 121.8 (2*C, 3,5-C_Py_), 128.8 (2*C, 2,6-C_Ph_), 129.3 (2*C, 3,4,5-C_Ph_), 132.4 (C_Ph_), 144.8 (C_Py_), 149.9 (2,6-C_Py_), 153.9 (C=N).

#### 3.1.2. General Procedure for the Synthesis of *E/Z*-GIZH-298

To the stirred suspension of 60% of the dispersion of NaH in mineral oil (8.80 g, 0.22 mol) in 15 mL of dry DMF was added dropwise to the solution of the corresponding (*E*- or *Z*-) 4-benzoylpyridine oxime (19.82 g, 0.1 mol) in 200 mL of dry DMF. After addition, the reddish-colored mixture was stirred for 2 h. Following that, 4-(2-Chloroethyl)morpholine (16.50 g, 0.11 mol) was added dropwise during 5 min. The mixture was then stirred overnight at room temperature. It was then poured into 1.5 L of distilled water and extracted with ethyl acetate (4 × 200 mL). The combined organic extract was washed with distilled water (3 × 300 mL) and the solvent was distilled off on a rotary evaporator. Ethyl acetate was added again to the residual 200 mL of ethyl acetate and distilled off on a rotary evaporator in order to remove the residual water. The resulting yellow-orange residue was dissolved in 100 mL of ethanol, and a solution of oxalic acid (9.00 g, 0.1 mol) in 25 mL ethanol was added. The solution was then kept for 1 h at room temperature. The precipitation of white solids was observed. It was then diluted with 500 mL of dry diethyl ether and placed in the refrigerator overnight. The precipitate was filtered off, washed with dry diethyl ether (2 × 200 mL), and dried in the air, affording the crude oxalate. The entire amount of the crude oxalate was dissolved in boiling ethanol (4 mL per 1 g for *Z*-oxalate, 16 mL per 1 g for *E*-oxalate), and the hot solution was filtered and left to crystallize for 24 h at room temperature. The precipitated crystals were filtered off, washed with ethanol (50 mL), dry diethyl ether (50 mL) and dried in an oven at 80 °C for 8 h. The products were obtained in the forms of white, finely crystalline powders.

**(*Z*)-phenyl(pyridin-4-yl)methanone *O*-(2-morpholinoethyl) oxime oxalate (*Z*-GIZH-298)**

White solid. Yield—80%. MP. = 159–160 °C. R*_f_* = 0.84 (chloroform:methanol = 10:1). ^1^H NMR (DMSO-*d6*, δ, ppm., *J*/Hz): 2.87 (t, 4H, *J*= 4.6 Hz, CH_2_(CH_2_-N-CH_2_)); 3.14 (t, 2H, *J*= 5.4 Hz, CH_2_-N); 3.54–3.89 (m., 4H, CH_2_-O-CH_2_); 4.41 (t, 2H, *J*= 5.4 Hz, CH_2_O); 7.30–7.38 (m., 2H, 3,5-H_Py_); 7.37–7.51 (m., 5H, H_Ph_); 8.66–8.79 (m., 2H, 2,6-H_Py_); 12.07 (br. s., 2H, COOH).

^13^C NMR (DMSO-*d6*, δ, ppm.): 52.2 (2*C, C-N-C), 55.3 (C-N), 64.2 (2*C, C-O-C), 69.9 (C-ON), 123.7 (2*C, 3,5-C_Py_), 127.6 (2*C, 2,6-C_Ph_), 129.2 (2*C, 3,5-C_Ph_), 130.7 (4-C_Ph_), 134.4 (C_Ph_), 141.2 (C_Py_), 150.3 (2,6-C_Py_), 155.9 (C=N), 163.9 (2*C, COOH).

LC–MS [M+H^+^] = 312 *m*/*z*.

IR (cm^−1^, KBr disc): 3424, 3041, 2965, 2906, 2874, 1721(br), 1635, 1614, 1498, 1459, 1265, 1144, 987.

UV-Vis (λ, nm, (lgε)): 194.0 (4.55), 237.0 (4.06), 259.5 (4.09).

**(*E*)-phenyl(pyridin-4-yl)methanone *O*-(2-morpholinoethyl) oxime oxalate (*E*-GIZH-298)**

White solid. Yield—53%. MP. = 165–168 °C, R*_f_* = 0.2 (DCM:methanol = 20:1). ^1^H NMR (DMSO-*d6*, δ, ppm., *J*/Hz): 2.73–2.95 (m., 4H, CH_2_-N-CH_2_); 3.04–3.19 (m, 2H, CH_2_N); 3.58–3.74 (m, 4H, CH_2_-O-CH_2_); 4.38–4.52 (m, 2H, CH_2_ON); 7.28–7.42 (m, 4H, H_Ar_), 7.48–7.56 (m, 3H, H_Ar_); 8.58–8.65, (m, 2H, H_Py_); 11.08 (br. s., 2H, COOH).

^13^C NMR (DMSO-*d6*, δ, ppm.): 52.5 (2*C, C-N-C), 55.5 (C-N), 64.4 (2*C, C-O-C), 70.4 (C-ON), 122.0 (2*C, 3,5-C_Py_), 129.0 (2*C, 2,6-C_Ph_), 129.1 (2*C, 3,5-C_Ph_), 129.9 (4-C_Ph_), 131.8 (C_Ph_), 143.1 (C_Py_), 150.5 (2,6-C_Py_), 156.1 (C=N), 163.6 (2*C, COOH).

LC–MS [M+H^+^] = 312 *m*/*z*.

IR (cm^−1^, KBr disc): 3452, 3055, 3035, 2952, 2867, 1723 (br), 1606, 1589, 1496, 1450, 990.

UV-Vis (λ, nm, (lgε)): 192.0 (4.58), 232.5 (3.94), 263.5 (3.98).

### 3.2. X-Ray Diffraction Analysis (XRD)

Single crystal X-ray analysis of *Z*-GIZH-298 was determined using a Bruker D8 Quest diffractometer (Bruker Scientific Instruments, Billerica, MA, USA) equipped with MoKα radiation (λ = 0.71073 Å) and operated in ω- and φ-scan modes at 100(2) K. The structural solution was carried out using the Olex2-1.5 software package [41] in combination with the SHELXT program [42], followed by refinement against F^2^ using the least-squares method with anisotropic displacement parameters for all non-hydrogen atoms [43]. All hydrogen atoms at carbon atoms were placed in calculated positions and refined within the riding model; all O–H hydrogen atoms were located from the electron-difference map and refined freely. The main crystallographic details and refinement parameters are listed in Supplementary Materials. CCDC 2455804 contains the supplementary crystallographic data for this paper. These data can be obtained free of charge via https://www.ccdc.cam.ac.uk/structures/ (accessed on 24 May 2026) or from the CCDC (12 Union Road, Cambridge CB2 1EZ, UK; Fax: +44 1223 336033; deposit@ccdc.cam.ac.uk).

### 3.3. HPLC

HPLC studies were carried out on an Infinity 1260 liquid chromatograph (Agilent Technologies, USA) with a gradient pump and a spectrophotometric detector with variable wavelength (software—Agilent OpenLAB CDS ChemStation Rev. B.04.03-SP2). The analysis was performed under conditions designed for the determination of related impurities on a steel chromatographic column, BDS Hypersil C18 250 × 4.6 mm, 5 μm (Thermo Scientific, Waltham, MA, USA). A mixture of 0.02 M buffer solution of dipotassium hydrogen phosphate, acetonitrile and methanol (500: 200: 200, by volume) with a pH of 7.45 ± 0.05 was used as the mobile phase. The flow rate of the mobile phase was 1.3 mL/min, the column temperature was 25 ± 0.5 °C, and the detection wavelength was 210 nm.

Additional HPLC analysis was performed on a stainless-steel chromatographic column, Zorbax Eclipse Plus C18 150 × 4.6 mm, 5 µm (Agilent Technologies, Santa Clara, CA, USA) to allow the eluate to be used for mass spectrometry analysis. A mixture of a 0.1% formic acid solution in water (*v*/*v*) and acetonitrile (85:15, *v*/*v*) was used as the mobile phase. The flow rate was 1.0 mL/min, the column temperature was 25 ± 0.5 °C, and the detection wavelength was 210 nm.

### 3.4. Mass Spectrometry

*Z*-GIZH-298 and *E*-GIZH-298 samples were dissolved in a mixture of 1% dimethyl sulfoxide/ 0.1% formic acid/ 48.95% acetonitrile/ 48.95% water (*v*/*v*), respectively, with a final concentration of 20 µg/mL. The impurity fraction collected on an LC column was evaporated until dry by lyophilization, and then dissolved in a mixture of 0.1% formic acid in acetonitrile/0.1% formic acid in water (*v*/*v*) with a final concentration of 1.5 µg/mL.

The analysis was performed using a syringe pump (flow rate—5 μL/min (for impurity fraction—10 μL/min)) with electrospray ionization (H-ESI mode) on a TSQ Altis triple quadrupole mass spectrometer (Thermo Fisher Scientific, USA). When registering positive ions, scanning was carried out in the total ion current mode (positive Q1 (TIC—total ion current, full mode), as well as in the “Product Ions” mode. “Xcalibur” software (v.4.2) was used for data registration and processing (Thermo Scientific, Waltham, MA, USA).

### 3.5. Study of Photostability of Z-GIZH-298

The photostability test was carried out in accordance with requirements of the ICH scientific guideline Q1B “Photostability testing of new active substances and medicinal products” [29] on the ATLAS SUNTEST CPS+ device (Atlas Material Testing Technology LLC, Mount Prospect, IL, USA) with a set of light filters to provide radiation that meets the ID 65 standard. The temperature inside the chamber was controlled using the ATLAS Sun Cool cooling system (ATLAS MTT, USA) and did not exceed 25 °C.

### 3.6. Maximal Electroshock Test

The study was performed on male outbred mice weighing 24–27 g. The animals were raised on the Stolbovaya farm and were kept in the vivarium facility with controlled light (12 h light-dark cycle) and ad libitum access to food and water in accordance with SP 2.2.1.3218-14 “Sanitary and epidemiological requirements for the device, equipment and maintenance of experimental and biological clinics (vivariums)” 29 August 2014, No 51. The experiments were conducted between 10 a.m. and 1 p.m. to exclude the influence of daily biorhythms. This study was carried out in accordance with the “Guide for preclinical drug research” for substances with anticonvulsant activity [44] and strictly with the Order of the European Convention for the protection of vertebrate animals (Strasbourg 1986); Order of the Ministry of Health of the Russian Federation 1 April 2016, No 199 N. All animal procedures were approved by the Biomedical Ethics Commission of the Federal Research Center for Innovator and Emerging Biomedical and Pharmaceutical Technologies, protocol №15 dated 15.09.2025. The anticonvulsant activity of the GIZH-298 isomers was evaluated using a model of primarily generalized seizures induced by MES according to the methodological recommendations [44]. Electroconvulsive seizures were induced using a certified RodentShocker RS apparatus, type 221 (Harvard Apparatus GmbH, Holliston, MA, USA). The animals received electrical stimulation via specialized corneal electrodes (mode 1: 250 V, 12 mA, 0.3 s). The seizure severity was assessed using a scoring system: 0—no response; 1—clonic seizures without loss of the righting reflex; 2—clonic seizures with loss of the righting reflex; 3—tonic-clonic seizures (clonus of the hind limbs with tonic extension of the forelimbs); 4—tonic seizures with extension of all four limbs; 5—tonic extension of all four limbs resulting in death. Test compounds were dissolved in physiological saline and administered orally 30 min prior to MES. Sodium valproate (valproic acid substance, Sigma), used as the reference drug, was dissolved in physiological saline and administered orally at a dose of 300 mg/kg under the same regimen. Efficacy was evaluated based on the reduction in seizure score, the increase in the number of animals without tonic seizures and the percentage of survivors relative to the corresponding control groups. The animals were assigned to six groups: (1) MES control; (2) and (3) *Z*-GIZH-298 at 60 mg/kg and 100 mg/kg, respectively; (4) and (5) *E*-GIZH-298 at 60 mg/kg and 100 mg/kg, respectively; and (6) sodium valproate at 300 mg/kg. Working solutions of the compounds were prepared in physiological saline in order for each mouse to receive 0.1 mL of solution per 10 g of body weight. Control animals were administered physiological saline at an equivalent volume. Statistical analysis was performed using MS Excel 2010 and BioStat 2009 (AnalystSoft Inc., Brandon, FL, USA). The normality of data distribution was assessed using the Shapiro–Wilk test. Differences between groups were analyzed using nonparametric tests: the Kruskal–Wallis test and Fisher’s exact test.

### 3.7. Genotoxicity Evaluation

The genotoxicity was evaluated in accordance with the recommendations in [34]. The mutagenicity was assessed using the Ames test with the Ames MPF™ PENTA I kit (Xenometrix AG, Allschwil, Switzerland) [34]. The testing was performed following the manufacturer’s instructions using the *S. typhimurium* strains TA98, TA100, TA1535, and TA1537, as well as a combination of *E. coli* pKM101 and uvrA strains, both with and without metabolic activation. For each concentration of the pharmaceutical substances *Z*-GIZH-298 and its *E*-isomer, the number of wells containing revertant colonies was determined. The obtained values were compared with the corresponding control using a two-tailed binomial test.

For the chromosomal aberration assay in mouse bone marrow cells, the pharmaceutical substance *Z*-GIZH-298 diluted in physiological saline was administered intraperitoneally to F1 CBA × C57Bl/6 hybrid mice: males received single doses of 40, 100, or 200 mg/kg with a 24 h exposure, and males and females received 40 mg/kg repeatedly (five administrations at 24 h intervals), with a sample collection 6 h after the final injection. Physiological saline was used as a negative control and was administered intraperitoneally to the control group in equivalent volumes. Mitomycin C was administered to male mice intraperitoneally as a single dose of 2.5 mg/kg served as a positive control. Cytogenetic preparations of femoral bone marrow cells were obtained using the standard air-dry technique [35]. Preparations were examined with a Standart-20 microscope (Carl Zeiss, Jena, Germany) under oil immersion at ×1000 magnification. Cells exhibiting chromatid gaps, chromosomal and chromatid breaks, and various types of exchanges were recorded [36]. For each animal (*n* = 5 per group), 100 metaphases were analyzed. Statistical evaluation (Fisher’s angular transformation, φ-test) was conducted by comparing the proportions of damaged metaphases between control and experimental groups.

DNA damage in mouse organs and tissues was assessed using the alkaline comet assay according to the recommendations in [37]. *Z*-GIZH-298 diluted in physiological saline was administered intraperitoneally to male F1 CBA × C57Bl/6 mice at doses of 40 and 200 mg/kg with exposure times of 3 and 24 h. Physiological saline was administered intraperitoneally in equivalent volumes to the negative control group. Methyl methanesulfonate administered intraperitoneally at 40 mg/kg with a 3 h exposure served as the positive control. The comet assay slides were analyzed using a Mikmed-2 12T epifluorescence microscope (LOMO, St. Petersburg, Russia) equipped with a high-resolution digital camera (VEC-335, EVS, St. Petersburg, Russia) at ×200 magnification. The images of DNA comets were processed using CASP 1.2.2 software [37,38,39]. The percentage of DNA in the comet tail (% tail DNA) was used as the indicator of DNA damage. For each animal (*n* = 5 per group), 100 DNA comets were analyzed. Statistical analysis was performed using Dunnett’s test for multiple comparisons and the Mann–Whitney U test.

## 4. Conclusions

Based on a series of experiments on the synthesis, purification, and characterization of the *E*- and *Z*-isomers of GIZH-298, the following conclusions can be drawn:

1. Both *E*- and *Z*-isomers of GIZH-298 could be synthesized and isolated in pure forms, owing to the possibility of separating the precursor oximes at the initial stage of synthesis.
2. The two isomers exhibit notable differences in their physicochemical characteristics. Significant variations in the chemical shifts of aromatic protons and carbons are observed both in ^1^H and ^13^C NMR spectra. IR and UV spectra show similar sets of absorption bands for both isomers, with minor differences. The melting point of the *E*-isomer is approximately 7 °C higher than that of the *Z*-isomer.
3. The *Z*-isomer is photosensitive and can undergo conversion to the *E*-form upon irradiation, as confirmed by both theoretical and experimental data. The *E*-isomer can also be accumulated during prolonged heating of the *Z*-isomer in polar solvents. At room temperature, the *Z*-isomer remains stable in aqueous and acidic media.
4. In the standard MES test for anticonvulsant activity, both isomers significantly suppress seizure activity; however, the *Z*-isomer demonstrates markedly higher efficacy than the *E*-isomer.
5. Neither the *Z*- nor the *E*-isomer exhibits genotoxicity.

The experimental and computational findings suggest that further development of *Z*-GIZH-298 as a potential antiepileptic agent should take into account the possibility of its conversion to the *E*-isomer, particularly under light exposure, necessitating storage conditions that exclude light.

The acceptance criterion for *E*-GIZH-298-related impurity content in the *Z*-GIZH-298 active pharmaceutical ingredient will be based on the partial retention of biological activity by the *E*-isomer, its safety considerations (lack of critical toxicity) and its presence in *Z*-GIZH-298 substance in accordance with the synthetic route.

## Figures and Tables

**Table 1:** Radioligand binding evaluation of *Z*-GIZH-298 to multiple biomolecular targets in vitro.

Biomolecular Targets	Type of Analysis	Radioligand	IC_50_, μM
D1 (h) ^1^	Antagonist radioligand	[^3^H]SCH 23390	>10
D2S (h) ^1^	Antagonist radioligand	[^3^H]-N-methylspiperone	>10
D2S (h) ^1^	Agonist radioligand	[^3^H]7-OHDPAT	>10
D3 (h)	Antagonist radioligand	[^3^H]-N-methylspiperone	>10
NMDA ^2^	Antagonist radioligand	[^3^H]MK-801(+)	>10
mGluII ^2^	Agonist radioligand	[^3^H]LY-354740	>10
AMPA ^1^	Agonist radioligand	[^3^H]AMPA	>10
5-HT_1A_ (h) ^1^	Agonist radioligand	[^3^H]8-OHDPAT	>10
5-HT_2A_^2^	Antagonist radioligand	[^3^H]ketanserin	>10
5-HT_2C_ (h) ^1^	Antagonist radioligand	[^3^H]mesulergine	>10
L-type Ca^2+^ channel (dihydropyridine binding site) ^1^	Antagonist radioligand	[^3^H]nitrendipine	>10
L-type Ca^2+^ channel (diltiazem binding site) ^1^	Antagonist radioligand	[^3^H]diltiazem	>10
L-type Ca^2+^ channel (verapamil binding site) ^1^	Antagonist radioligand	[^3^H]D888	>10
N-Ca^2+^- channel ^1^	Antagonist radioligand	[^125^I]ω-conotoxin GVIA	>10
Na^+^- channel ^1^	Antagonist radioligand	[^3^H]batrachotoxin	>10
D-transporter (h) ^1^	Antagonist radioligand	[^3^H]BTCP	>10
5-HT transporter (h) ^1^	Antagonist radioligand	[^3^H]imipramine	>10

^1^ Data of Eurofins Discovery. ^2^ Data of the Federal Research Center for Innovators and Emerging Biomedical and Pharmaceutical Technologies.

**Table 2:** Completed stages of *Z*-GIZH-298 preclinical development as a potential anticonvulsant.

Specific activity of *Z*-GIZH-298	MES test	Active (single and subchronic i.p. administration, mice and rats) [15]
	Model of secondarily generalized seizures in a rat with a chronic cobalt-induced epileptogenic focus	Active (single i.p. administration, rats) [8]
	Model of epileptic status induced by homocysteine thiolactone in rats with a chronic epilepsy model	Active (single i.p. administration, rats) [8]
Additional effects of *Z*-GIZH-298	Anxiolytic activity	Active upon subchronic administeration in the elevated plus-maze test (i.p., mice) [13]
	Analgesic activity	Active (p.o.) across a broad range of models, including the formalin test, capsaicin test, von Frey test, acetic acid writhing test, and tail-flick test in mice [14]
Assessment of possible side effects of *Z*-GIZH-298	Assessment of spatial working memory using the Y-shaped maze apparatus.	Does not affect cognitive functions (subchronic i.p. administration, mice) [13]
	Rotating Rod and Pull-Up Tests	Does not induce neurological deficits (i.p. administration, mice, rats) [8]
Toxicity Assessment of *Z*-GIZH-298	Acute toxicity in outbred mice (i.p.)	LD_50_ = 299.6 mg/kg (females) LD_50_ = 302.3 mg/kg (males) [16]
	Acute toxicity in outbred mice (p.o.)	LD_50_ = 356 mg/kg (females) LD_50_ = 438 mg/kg (males) [17]
Pharmacokinetics of *Z*-GIZH-298	Method for quantitative determination in blood plasma	A selective and sensitive HPLC-MS method for quantification of *Z*-GIZH-298 in rat plasma was developed and validated [18]
	Pharmacokinetics after a single i.v. dose of 60 mg/kg (rats)	GIZH-298 was detectable in plasma for 4 h. The apparent initial concentration (C_0_) was 1402 ng/mL. The elimination half-life (t_1_/_2_,el) was 0.48 h. The apparent volume of distribution (Vd) was 8.71 L/kg [19]
	Pharmacokinetics after a single p.o. dose of 60 mg/kg (rats)	The plasma elimination half-life (t_1_/_2_) was 0.66 h. Tissue-to-plasma concentration ratios were: liver 9.98, kidney 5.53, spleen 3.79, skeletal muscle 1.72, and brain 0.92. The absolute bioavailability of *Z*-GIZH-298 was 64.5% [20]
	Metabolism	The main metabolites in plasma and liver homogenates were identified as molecules with *m*/*z* 326 (hydroxylated derivatives) and *m*/*z* 328 (oxomorpholine derivative) [21]

**Table 3:** Results from the study of electrospray ionization of target compounds.

Ions	Target Compound		
	*Z*-GIZH-298	*E*-GIZH-298	Impurity Fraction of *Z*-GIZH-298, Collected on an LC Column
Molecular (precursor), *m*/*z*	312	312	312
Product, *m*/*z*	114, 181	114, 181	114, 181

**Table 4:** Analysis of the configurational stability of *Z*-GIZH-298.

№	Solvent	Time, h	Boiling point, °C	% of *E*-Isomer
1	H_2_O	5	100	5
2	H_2_O	24	100	9
3	dioxane	5	101	4
4	toluene	5	110	0

**Table 5:** Energy and geometric parameters for isomers of the model molecule and transition states (TS) of transitions between them in the ground and excited states.

Parameters	Calculation Results			
	*Z*-Isomer ^a^	*E*-Isomer ^a^	TS (*S*_0_) ^a^	TS (*S*_1_) ^b^
Relative energy [kcal/mol]	1.98	0	60.27	100.46
C-N [Å]	1.29757	1.29824	1.25986	1.44634
N-O [Å]	1.37259	1.36949	1.30263	1.35491
ONC [°]	113.277	113.284	177.674	37.409
NCC [°]	124.155	34.422	120.719	115.596
NOC [°]	1.41817	109.314	113.022	34.687
CCNO [°]	1.010	177.996	93.767	93.766
CNOC [°]	180.446	179.920	86.010	85.114
Wavelength (calculated), [nm] ^c^	251	256	-	-

^a^ Geometry optimized at SA-CASSCF(14,14)/ANO-RCC-VDZP/PCM(toluene); relative energy obtained at multistate-CASPT2/ANO-RCC-VDZP/PCM(toluene) as a single-point calculation on the CASSCF-optimized geometry. ^b^ Geometry optimized at TD-BHandHLYP/def2-TZVPD/PCM(toluene); relative energy obtained at multistate-CASPT2/ANO-RCC-VDZP/PCM(toluene) as a single-point calculation on the TD-BHandHLYP-optimized geometry. ^c^ Vertical excitation wavelength computed at the multistate-CASPT2/ANO-RCC-VDZP/PCM(toluene) level on the SA-CASSCF-optimized geometry of the corresponding isomer.

**Table 6:** Effect of *E*- and *Z*-isomers of GIZH-298 on seizures in the MES test (electrical parameters: 250 V/12 mA, 0.3 s).

Animal Group	Dose mg/kg	Severity of Seizure (Score)	Number of Mice with Tonic Extension, Abs. Units (%)	Number of Mice with Tonic Extension and Death, Abs. Units (%)
Control NaCl 0.9%	-	4 4 ÷ 4 2 ÷ 5	10/11 (91)	2/11 (18)
*Z*-GIZH-298	60	3 2 ÷ 4 2 ÷ 4	4/11 (36) ^&^	0/11 (0)
	100	2 * 1 ÷ 2 1 ÷ 3	0/11 (0) ^&&&^	0/11 (0)
*E*-GIZH-298	60	4 1 ÷ 5 0 ÷ 5	7/11 (64)	3/11 (27)
	100	2 2 ÷ 4 1 ÷ 4	3/11 (27) ^&^	0/11 (0)
Sodium valproate	300	2 * 1 ÷ 3 0 ÷ 4	2/11 (18) ^&&^	0/11 (0)

Notes: Median, lower–upper quartiles, and minimum–maximum values are shown. * *p* < 0.01 vs. control (Kruskal–Wallis test followed by Dunn’s multiple-comparison procedure). ^&^ *p* < 0.05; ^&&^ *p* < 0.01; ^&&&^ *p* < 0.001 vs. control (Fisher’s exact test with Benjamini–Hochberg correction for multiple comparisons).

**Table 7:** Effect of *Z*-GIZH-298 on the level of chromosomal aberrations in mouse bone marrow cells in vivo.

Group	Per 100 Cells					Metaphases with Aberrations (%, m ± SE)
	Gaps	Chromatid Breaks	Chromosome Breaks	Chromatid Exchanges	MD *	
male mice						
Control	0.2	1.2	0	0	0	1.4 ± 0.5
Mitomycin C 2.5 mg/kg	2.4	27.6	0.2	1.4	4.2	24.2 + 1.9 **
*Z*-GIZH-298 40 mg/kg, once	0	0.4	0	0	0	0.4 ± 0.3
*Z*-GIZH-298 100 mg/kg, once	0.4	0.4	0	0.2	0	1.0 ± 0.4
*Z*-GIZH-298 200 mg/kg, once	0	1.0	0	0.2	0	1.2 ± 0.5
*Z*-GIZH-298 40 mg/kg, 5-fold	0.4	1.2	0	0	0	1.4 ± 0.5
female mice						
Negative control	0.2	0.8	0	0	0	1.0 ± 0.5
*Z*-GIZH-298 100 mg/kg, 5-fold	0.2	0.4	0	0	0	0.6 ± 0.3

* Cells with multiple chromosomal damages (≥5 aberrations per metaphase); **—*p* < 0. compared to the control (Fisher’s φ (phi-star) criterion).

**Table 8:** Effect of *Z*-GIZH-298 on DNA damage in mouse organs and tissues in vivo.

Experimental Group	% DNA in the Tail			
	Bone Marrow	Liver	Kidneys	Spleen
3 h exposure				
Control	1.7 ± 0.6	1.7 ± 0.5	2.9 ± 0.5	0.8 ± 0.2
Methyl methanesulfonate 40 mg/kg	7.3 ± 1.0 *	13.0 ± 0.9 *	12.3 ± 1.3 *	6.4 ± 0.7 *
*Z*-GIZH-298 40 mg/kg	1.7 ± 0.4	2.7 ± 0.5	2.5 ± 0.7	0.7 ± 0.1
*Z*-GIZH-298 200 mg/kg	1.7 ± 0.9	2.4 ± 1.2	2.0 ± 1.1	0.7 ± 0.2
24 h exposure				
Control	1.5 ± 0.6	2.0 ± 0.6	1.6 ± 0.7	1.6 ± 0.5
GIZH-298 40 mg/kg	2.2 ± 0.3	2.6 ± 0.5	1.5 ± 0.5	1.6 ± 0.5
GIZH-298 200 mg/kg	1.5 ± 0.8	2.2 ± 1.1	1.7 ± 0.6	1.6 ± 0.3

*—*p* < 0.001 compared to control (Mann–Whitney U test).

## Data Availability

The data presented in this study are available on request from the corresponding author.

## Supplementary Materials

The following supporting information can be downloaded at: https://www.mdpi.com/article/10.3390/molecules31111872/s1, ^1^H, ^13^C spectra of (*Z*)-phenyl(pyridin-4-yl)methanone oxime (**2a**); ^1^H, ^13^C spectra of (*E*)-phenyl(pyridin-4-yl)methanone oxime (**2b**); ^1^H, ^13^C, COSY, DEPT, HSQC and NOESY spectra of *E*- and *Z*-GIZH-298; Table S1: Main crystallographic details and refinement parameters for structures; Table S2: Parameters of intermolecular hydrogen bonds in *Z*-GIZH-298; HPLC chromatograms of *E*- and *Z*-GIZH-298.

## Abbreviations

The following abbreviations are used in this manuscript:

AMPA	α-amino-3-hydroxy-5-methyl-4-isoxazolepropionic acid receptor
CA	carbonic anhydrase
Cav	voltage-gated calcium channels
CB	cannabinoid receptor
DMSO	dimethyl sulfoxide
GABA	γ-aminobutyric acid
GABA-T	GABA transaminase
GAD	glutamic acid decarboxylase
GAT	GABA transporter
HPLC	high performance liquid chromatography
ICH	The International Council for Harmonisation of Technical Requirements for Pharmaceuticals for Human Use
Kv	voltage-gated potassium channel
Nav	voltage-gated sodium channels
NMDA	*N*-methyl-D-aspartate
mGluR	metabotropic glutamate receptors
SV2A	synaptic vesicle glycoprotein 2A
