# Supplementary material for: E/Z Isomerism of the Potential Antiepileptic Drug GIZH-298
Source: Molecules. 2026 May 29;31(11):1872. doi: 10.3390/molecules31111872 (PMC13258319; doi:10.3390/molecules31111872)
Supplement: Supplementary file 1 [file molecules-31-01872-s001.zip › molecules-4300478-supplementary.pdf]

Supporting Information

E/Z isomerism of the potential antiepileptic drug GIZH-298

G.V. Mokrov[1], S.A. Litvinova[2], V.E. Biryukova[1], T.A. Voronina[2], S.V. Shorunov[1], A.G. Rebeko[1], T.Yu. Vorobyova[1], L.N. Grushevskaya[3], M.S. Sergeeva[3], D.N. Kuznetsov[3], O.Yu. Kravtsova[4], E.A. Nikitina[2], N.A. Gladysheva[2], A.K. Zhanataev[5], A.D. Durnev[5], T.V. Losev[6], K.M. Malashkeevich[6], M.G Medvedev[6], M.M. Islamov[7], K.A. Lysenko[7], V.L. Dorofeev[3,8]

[1]Department of Medicinal Chemistry. Federal Research Center for Innovator and Emerging Biomedical and Pharmaceutical Technologies, Baltiyskaya 8, 125315, Moscow, Russia

[2]Department of Neuropsychopharmacology. Federal Research Center for Innovator and Emerging Biomedical and Pharmaceutical Technologies, Baltiyskaya 8, 125315, Moscow, Russia

[3]Laboratory of Standardization and Quality Control. Federal Research Center for Innovator and Emerging Biomedical and Pharmaceutical Technologies, Baltiyskaya 8, 125315, Moscow, Russia

[4]Pharmacokinetics Laboratory. Federal Research Center for Innovator and Emerging Biomedical and Pharmaceutical Technologies, Baltiyskaya 8, 125315, Moscow, Russia

[5]Department of Toxicology, Federal Research Center for Innovator and Emerging Biomedical and Pharmaceutical Technologies, Baltiyskaya 8, 125315, Moscow, Russia

[6]Group of Theoretical Chemistry, №24, Zelinsky Institute of Organic Chemistry, Russian Academy of Sciences, Leninsky Prospekt 47, 119991 Moscow, Russia

[7]Faculty of Chemistry, Lomonosov Moscow State University, Leninskie Gory 1/3, 119991 Moscow, Russia

[8]General Director. Federal Research Center for Innovator and Emerging Biomedical and Pharmaceutical Technologies, Baltiyskaya 8, 125315, Moscow, Russia

Corresponding authors:

G.V. Mokrov. Tel: +79261856846; E-mail address: mokrov_gv@academpharm.ru

V.E. Biryukova. Tel: +79775623224; E-mail address: biryukova_ve@academpharm.ru

Table of Contents

1. ^{1}H, ^{13}C spectra of ***(Z)*-phenyl(pyridin-4-yl)methanone oxime (2a)** 3

2. ^{1}H, ^{13}C spectra of ***(E)*-phenyl(pyridin-4-yl)methanone oxime (2b)** 4

3. ^{1}H, ^{13}C, COSY, DEPT, HSQC and NOESY spectra of ***E*-** and ***Z*-GIZH-298** 5

4. Table S1. Main crystallographic details and refinement parameters for structures. 12

5. Table S2. Parameters of intermolecular hydrogen bonds in ***Z-GIZH-298*** 13

6. HPLC chromatograms of ***E*-** and ***Z*-GIZH-298** 14

1. ^{1}H, ^{13}C spectra of *(Z)*-**phenyl(pyridin-4-yl)methanone oxime (2a)**

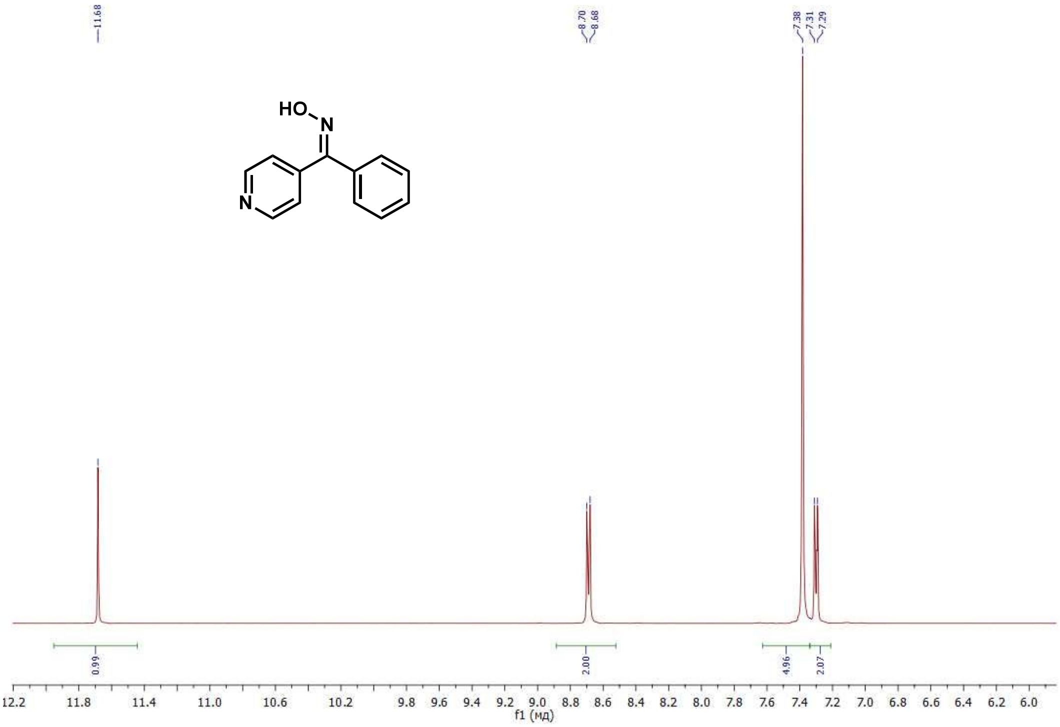

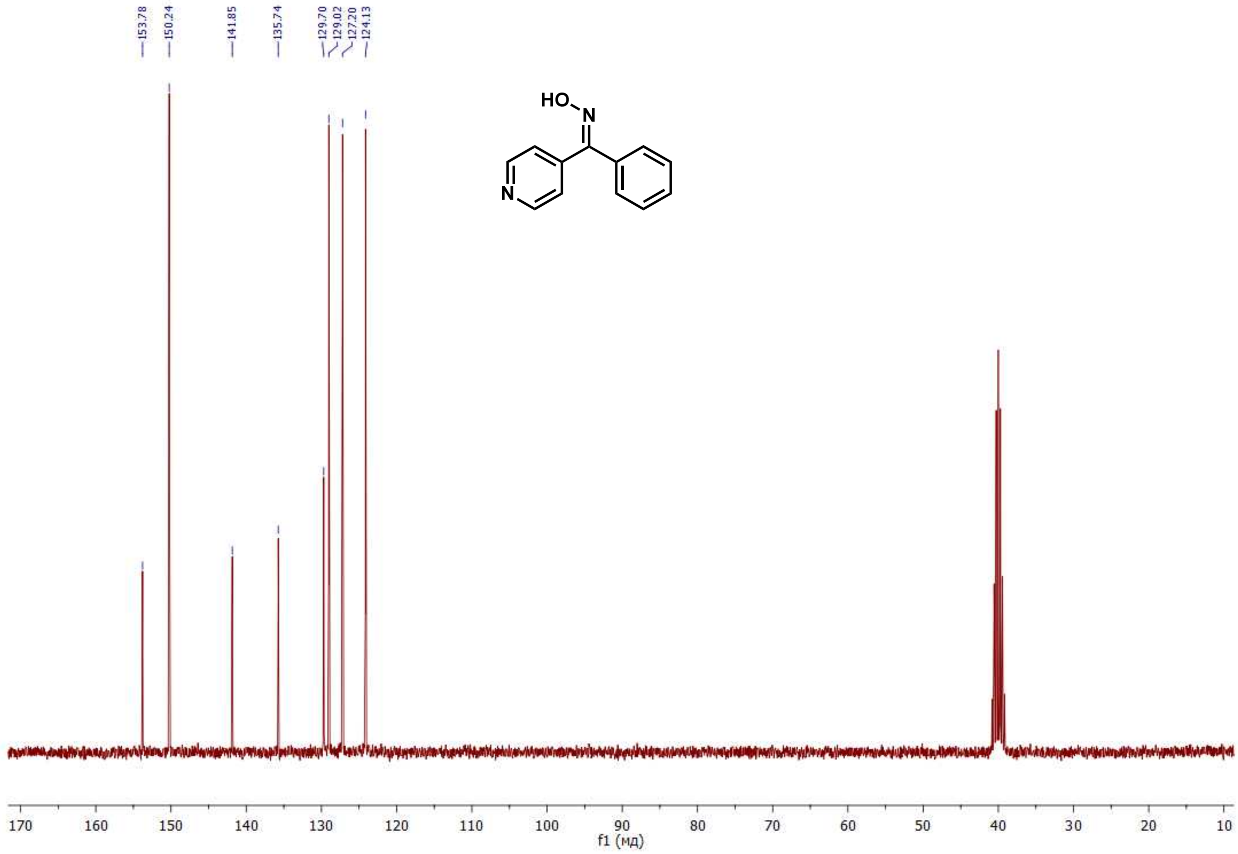

2. ¹H, ¹³C spectra of ***(E)*-phenyl(pyridin-4-yl)methanone oxime (2b)**

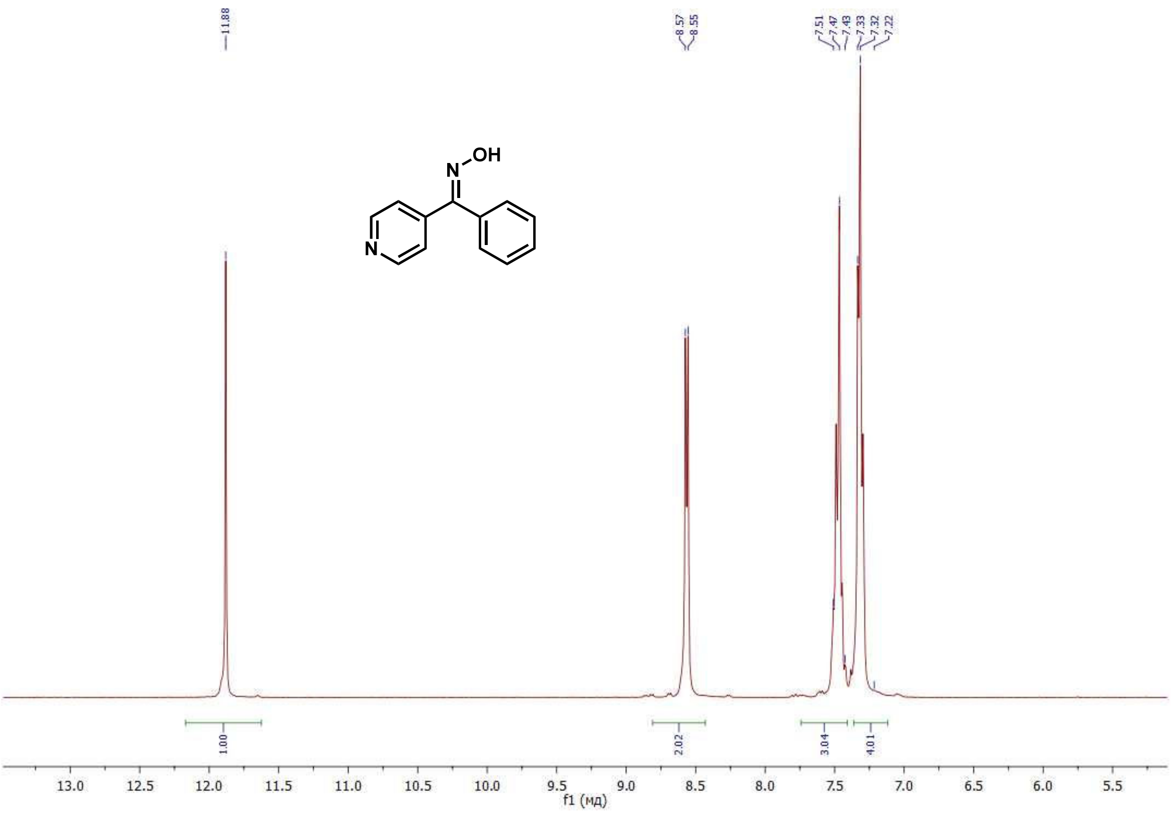

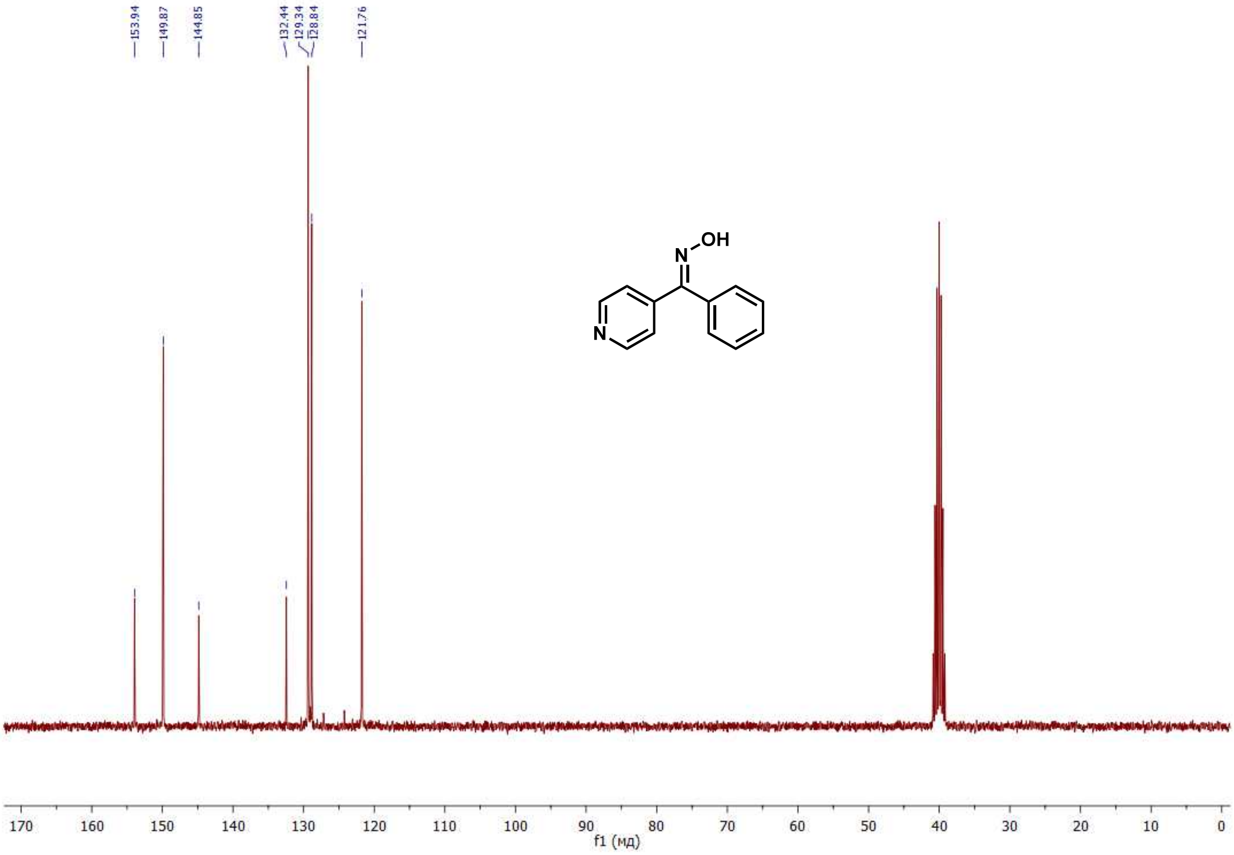

3. ^{1}H, ^{13}C, COSY, DEPT, HSQC and NOESY spectra of ***E*-** and ***Z*-GIZH-298**

***E*-GIZH-298** ((*E*)-phenyl(pyridin-4-yl)methanone *O*-(2-morpholinoethyl) oxime)

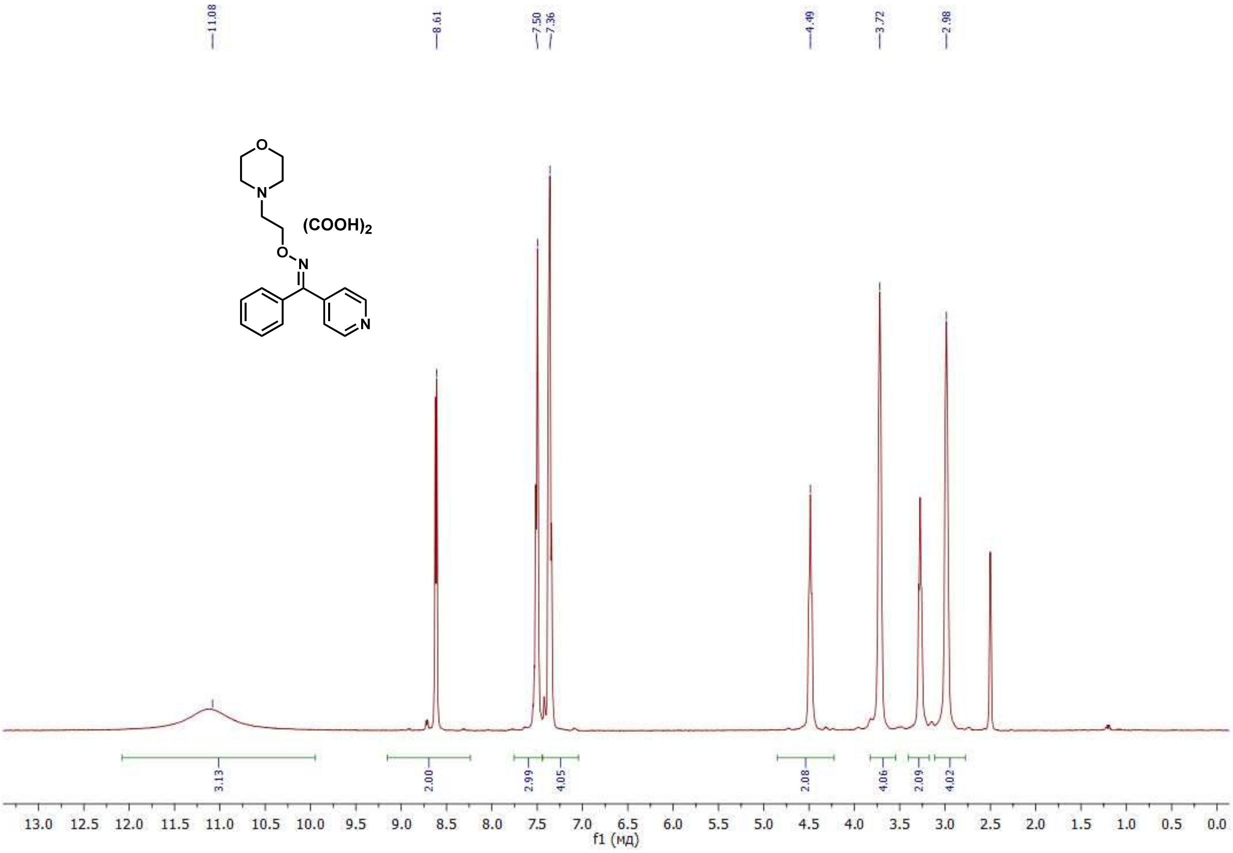

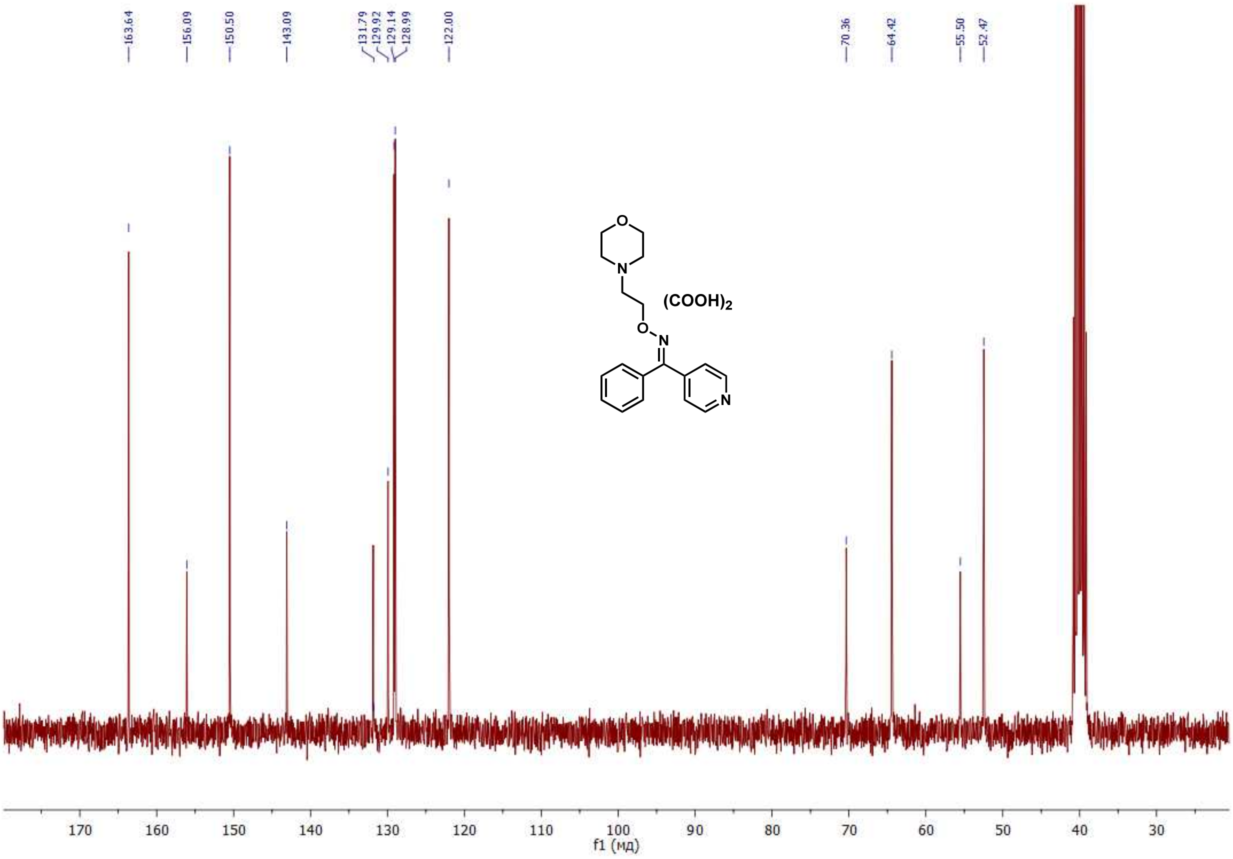

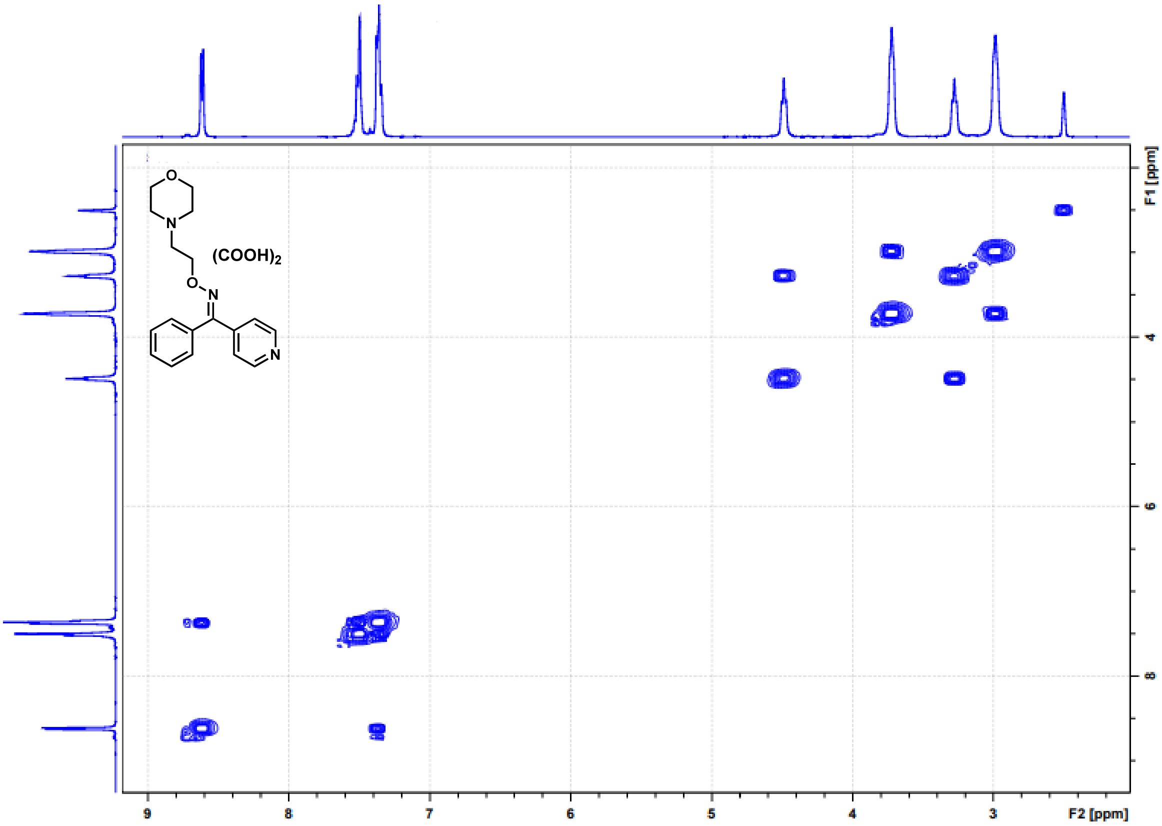
(COOH)2
O
N
F1 [ppm]
F2 [ppm]
9
8
7
6
5
4
3

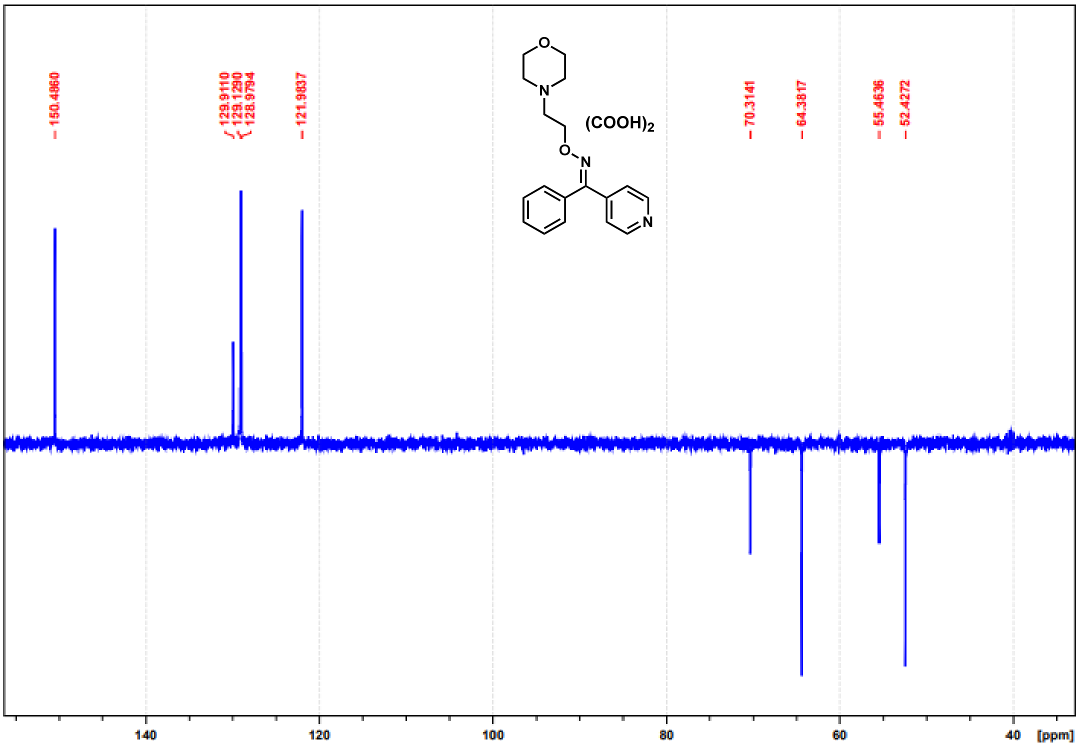
150.4860
129.9110
129.1290
128.9794
121.9837
70.3141
64.3817
55.4636
52.4272
(COOH)2
O
N
140
120
100
80
60
40
[ppm]

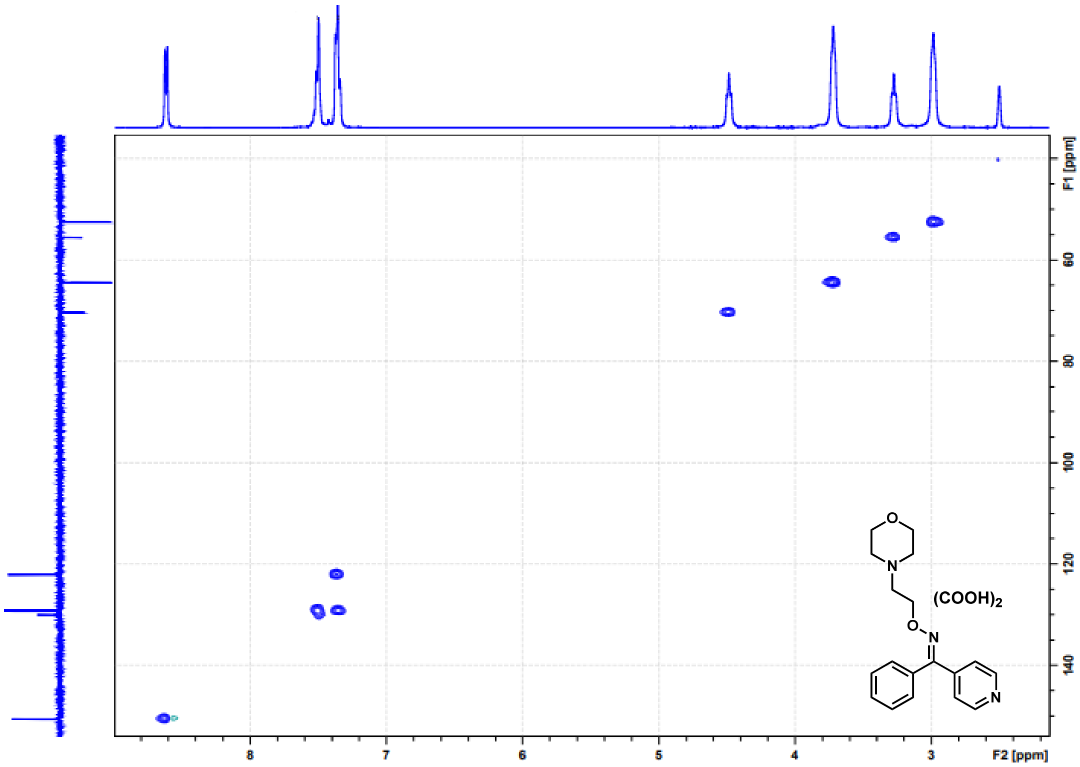
F1 [ppm]
F2 [ppm]
(COOH)2

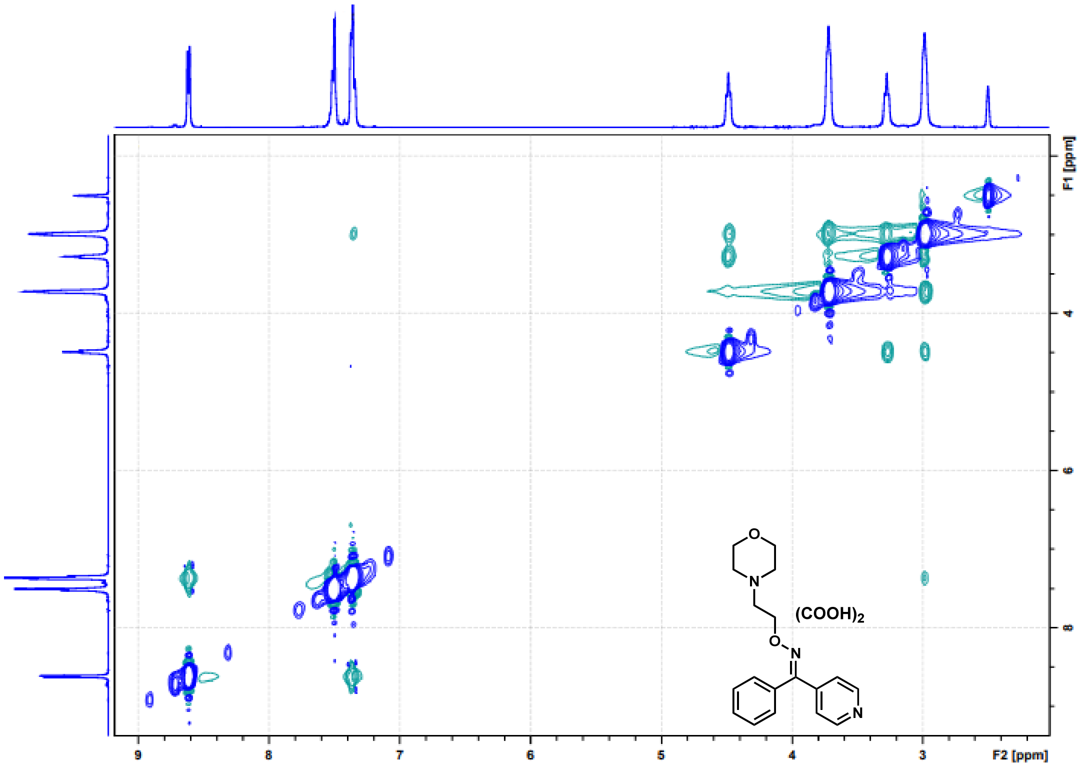
F1 [ppm]
F2 [ppm]
(COOH)2

Z-GIZH-298 ((*Z*)-phenyl(pyridin-4-yl)methanone *O*-(2-morpholinoethyl) oxime)

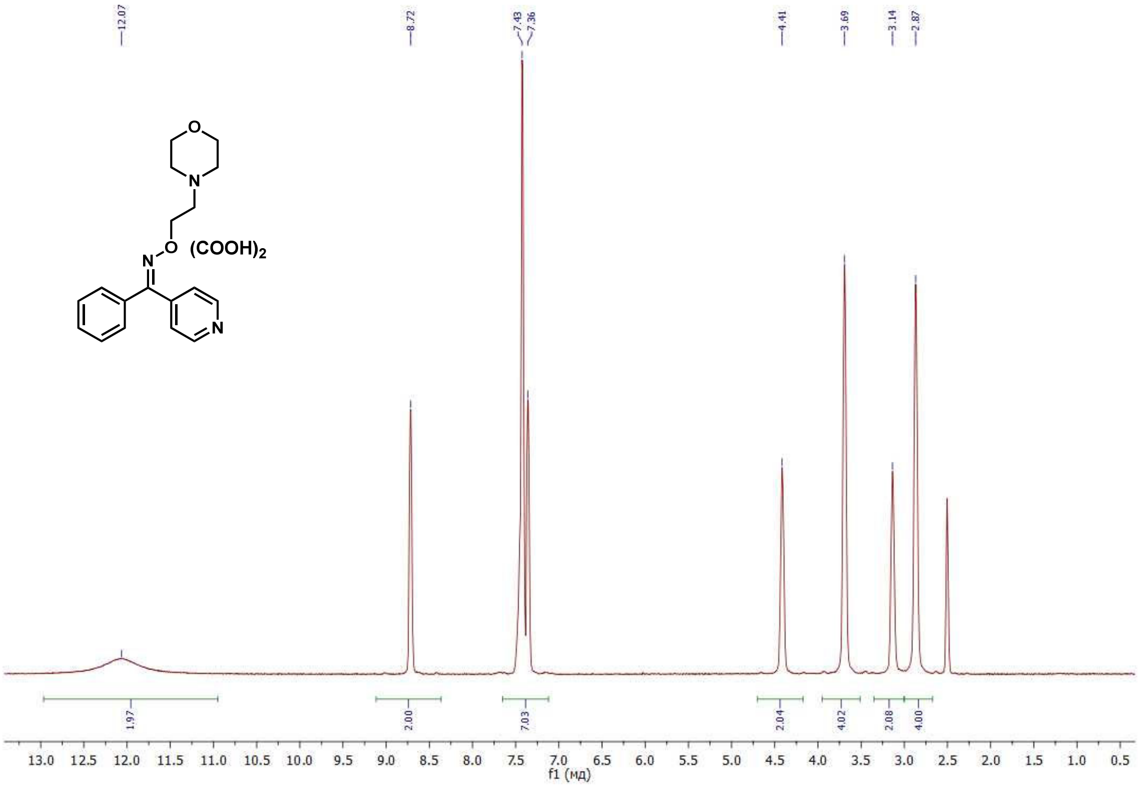

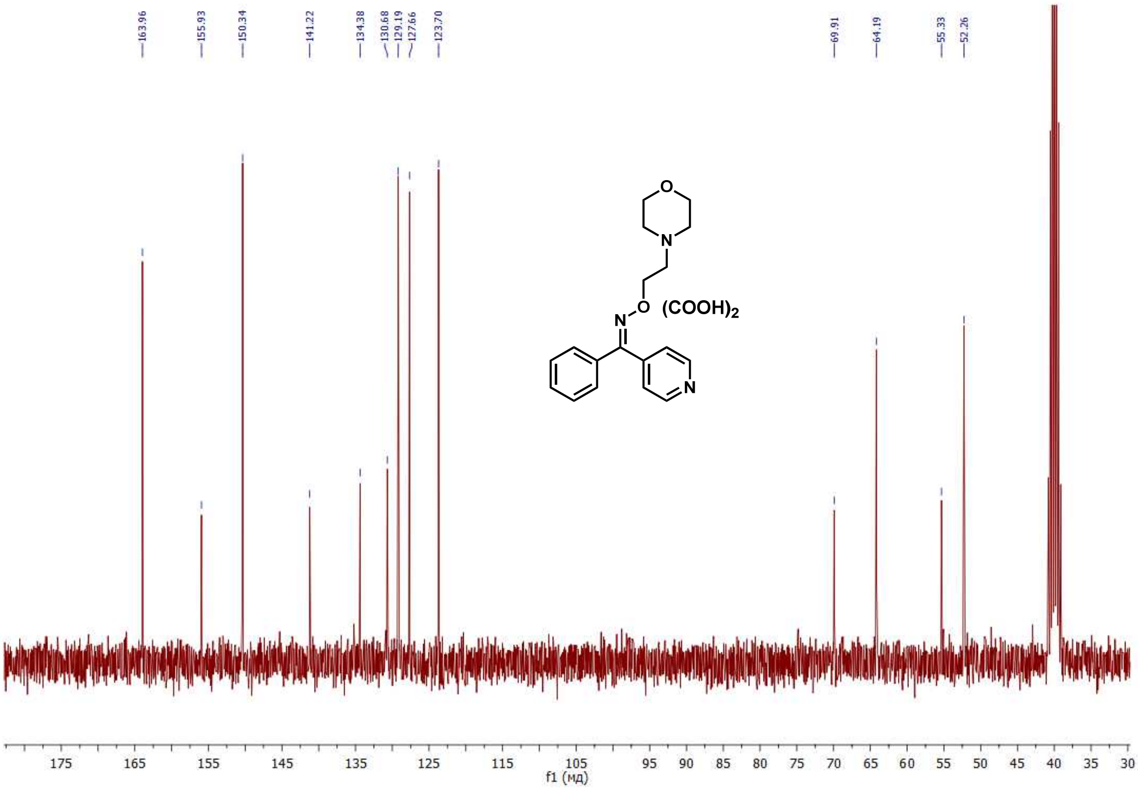

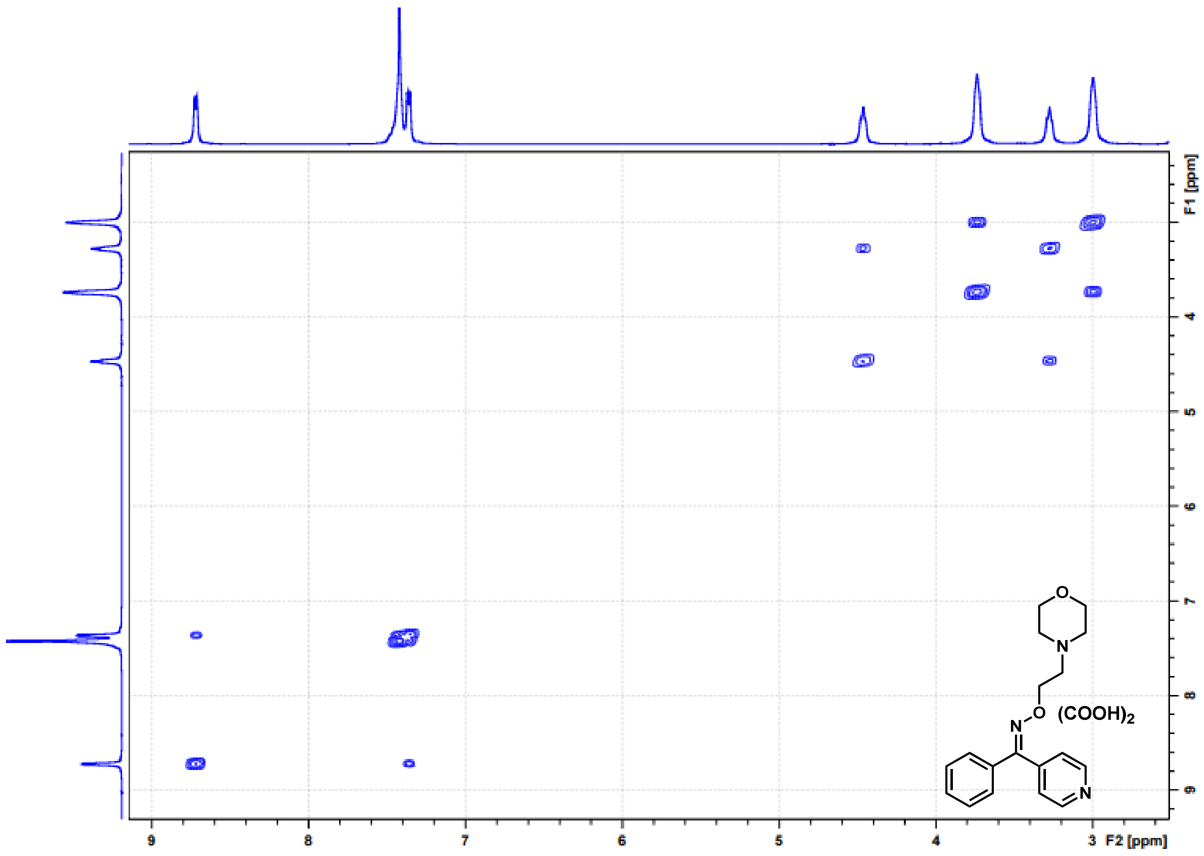
F1 [ppm]
F2 [ppm]
(COOH)2
N
O
N
O
N
9
8
7
6
5
4
3

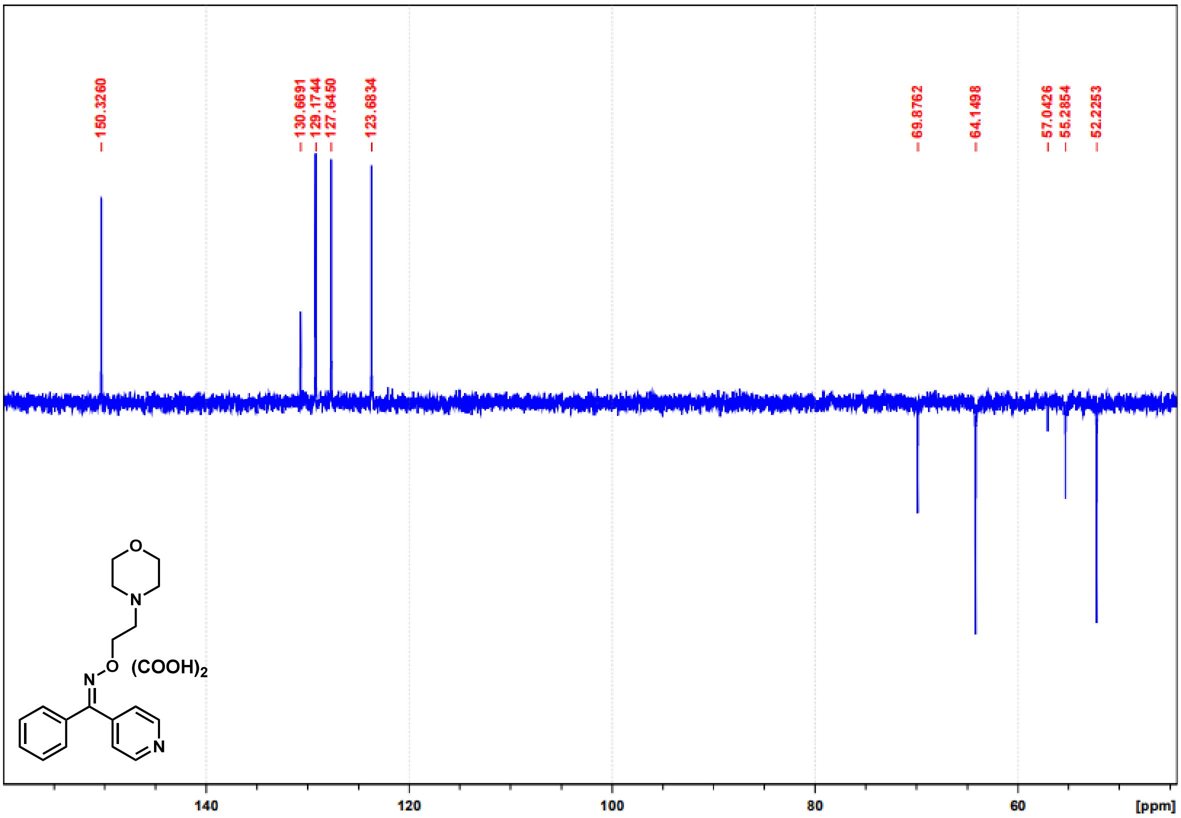

150.3260
130.6691
129.1744
127.6450
123.6834
69.8762
64.1498
57.0426
55.2854
52.2253
140
120
100
80
60
[ppm]
(COOH)2

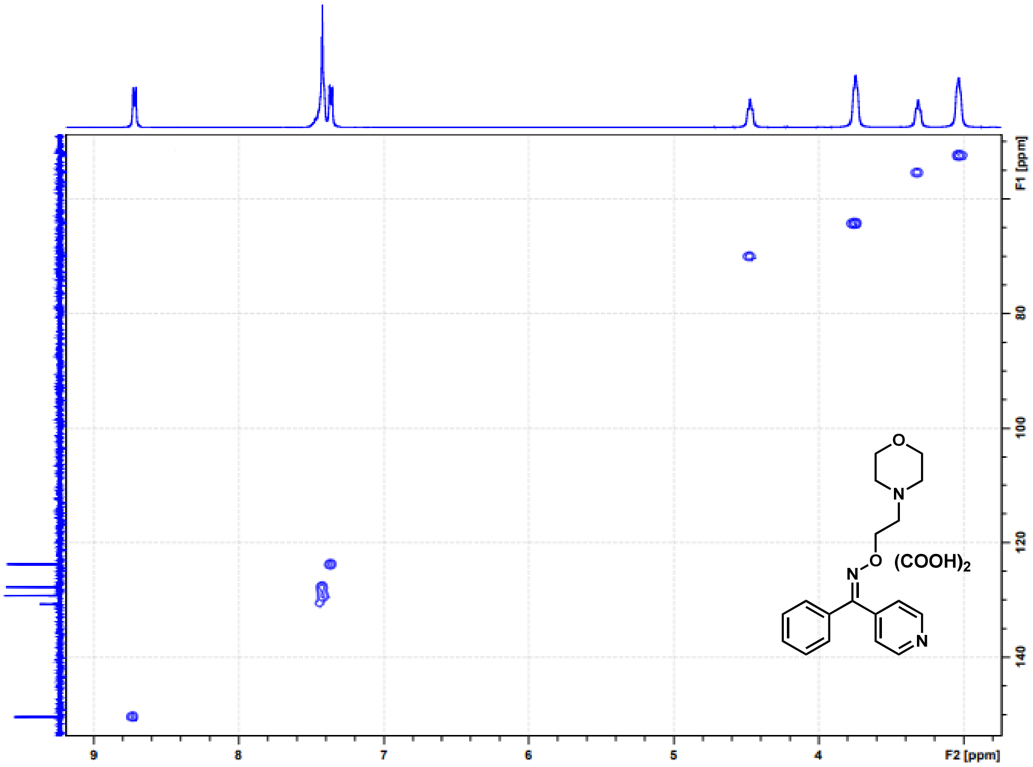

F1 [ppm]
80
100
120
140
9
8
7
6
5
4
F2 [ppm]
(COOH)2

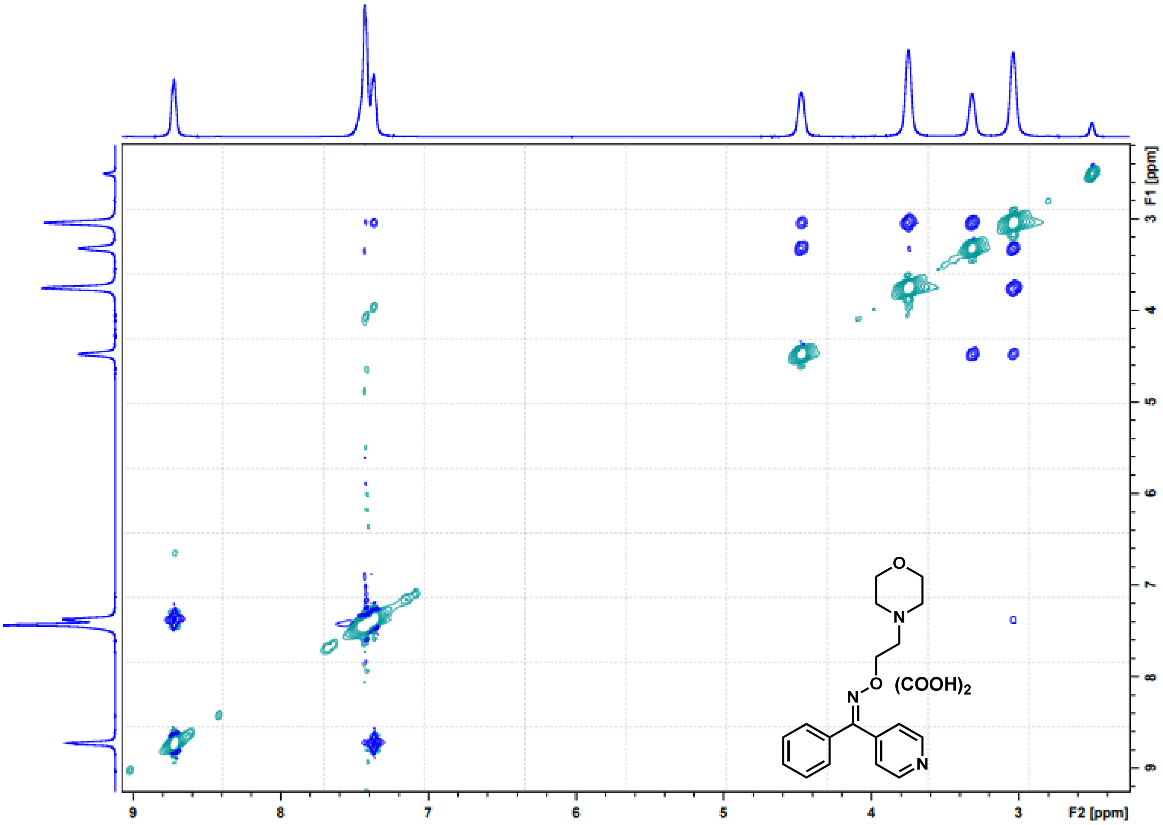

F1 [ppm]
F2 [ppm]
(COOH)2

4. Table S1. Main crystallographic details and refinement parameters for structures.

Compound	GIZH-298
CCDC	2421721
Empirical formula	$C_{20}H_{23}N_3O_6$
Formula weight (g·mol^{-}	401.41
Crystal system	Monoclinic
Space group	$P2_1/c$
a (Å)	8.2837(3)
b (Å)	22.1413(7)
c (Å)	10.8615(4)
α (deg)	90
β (deg)	109.5790(10)
γ (deg)	90
V (Å^3)	1876.94(11)
Z	4
D_{calc} (g·cm^{-3})	1.421
θ_{min}-θ_{max} (deg)	2.19-30.00
μ (mm^{-1})	0.11
Reflections/ Reflection unique number	19891/5461
Reflections with $I > 2\sigma(I)$	4452
R_{int}	0.0499
GooF	1.023
R_1, wR_2 ($I > 2\sigma(I)$)	0.0454, 0.1068
R_1, wR_2 (all data)	0.0564, 0.1145

5. Table S2. Parameters of intermolecular hydrogen bonds in ***Z-GIZH-298***

**Interaction**	**D–H, Å**	**H···A, Å**	**D···A, Å**	**D–H–A, deg.**
		**1**		
N3–H3···O3 (x, y, z)	0.90(2)	2.30(2)	2.952(2)	130(1)
N3–H3···O6 (x, y, z)	0.90(2)	2.02(2)	2.850(2)	154(1)
O3–H3A···O5 (x, ½-y, ½+z)	1.24(3)	1.21(3)	2.452(1)	175(2)
C7–H7···O3 (1-x, 1-y, 1-z)	0.97(2)	2.51(2)	3.379(2)	149(1)
C13–H13A···O6 (x, y, z)	0.99(2)	2.57(2)	3.308(2)	131(1)
C14–H14A···O4 (-1+x, y, z)	0.94(2)	2.52(5)	3.341(2)	146(7)
C15–H15A···O4 (-1+x, y, z)	0.98(2)	2.45(2)	3.343(2)	152(1)
C15–H15B···O6 (x, ½-y, ½+z)	0.98(2)	2.45(2)	3.382(2)	159(1)
C18–H18A···O5 (-1+x, y, z)	0.97(2)	2.58(2)	3.371(2)	139(1)
C18–H18B···O4 (-1+x, ½-y, - ½+z)	0.98(2)	2.48(2)	3.271(2)	137.5(1)

6. HPLC chromatograms of ***E***- and ***Z*-GIZH-298**

***(E)*-phenyl(pyridin-4-yl) methanone*O*-(2-morpholinoethyl) oxime oxalate (*E*-GIZH-298)**

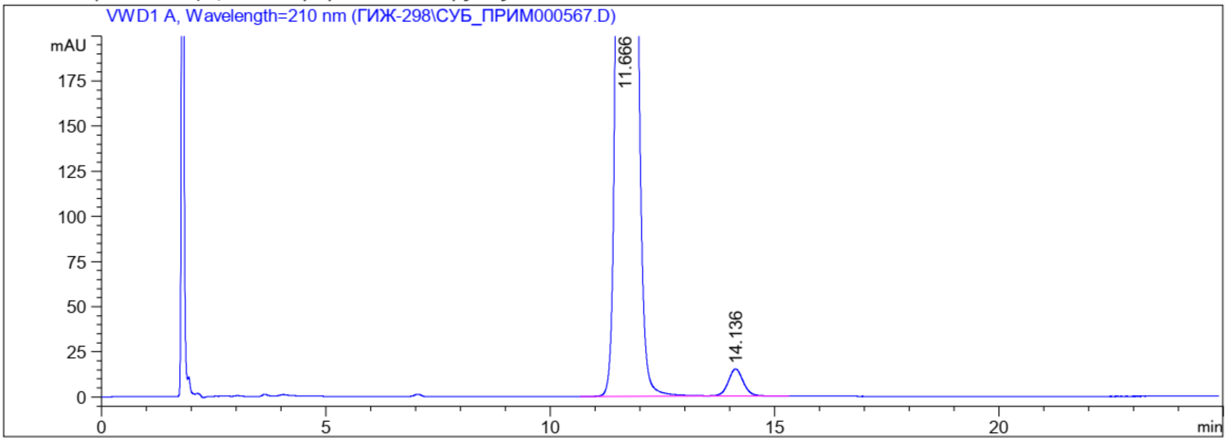


```
=====================================================================
                    Отчет Относительных Площадей
=====================================================================

Сортировка  :      Сигнал
Множитель          :      1.0000
Разбавление             :      1.0000
Применять Множитель & Разбавление к ISTD


Сигнал 1: VWD1 A, Wavelength=210 nm

Пик RetTime  Тип  Ширина  Площадь    Высота     Площадь
  #   [мин]         [мин]   [mAU*s]     [mAU]        %
----|-------|----|-------|----------|----------|--------|
   1  11.666 BV     0.2911 3.03436e4  1586.46631  98.8401
   2  14.136 VB     0.3619  356.07565   15.05955   1.1599

Всего :                   3.06996e4  1601.52586
```

(*Z*)-phenyl(pyridin-4-yl)methanone *O*-(2-morpholinoethyl) oxime oxalate (*Z*-GIZH-298)

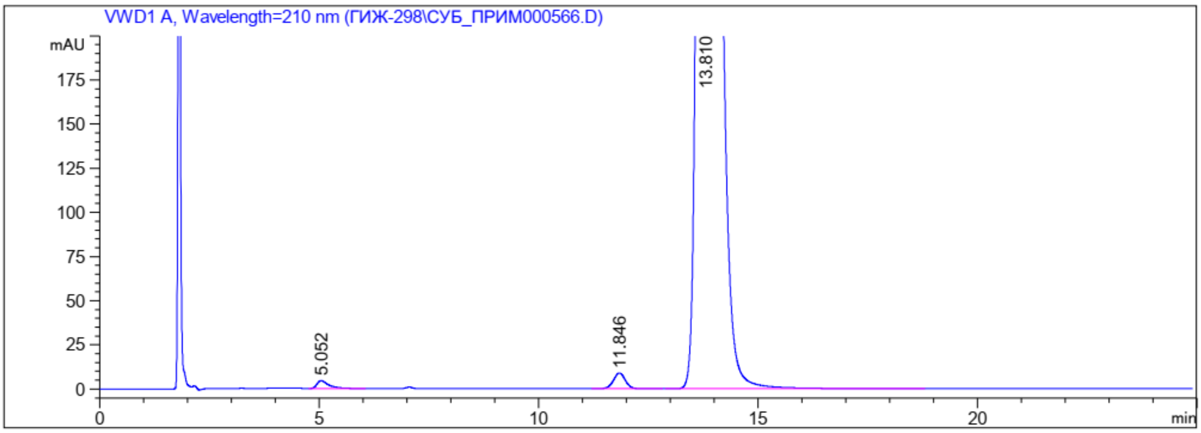


```
======================================================================
                    Отчет Относительных Площадей
======================================================================

Сортировка   :       Сигнал
Множитель            :       1.0000
Разбавление               :       1.0000
Применять Множитель & Разбавление к ISTD


Сигнал 1: VWD1 A, Wavelength=210 nm

Пик RetTime  Тип  Ширина  Площадь    Высота     Площадь
  #   [мин]        [мин]   [mAU*s]     [mAU]        %
----|-------|----|-------|----------|----------|--------|
   1   5.052 VB    0.2787   86.64606    4.49919   0.2281
   2  11.846 BB    0.2943  170.51877    8.94924   0.4490
   3  13.810 BB    0.3450 3.77226e4  1624.16553  99.3229

Всего :                  3.79797e4  1637.61396
```

(Z)-phenyl(pyridin-4-yl)methanone O-(2-morpholinoethyl) oxime oxalate (Z-GIZH-298) batch 260324

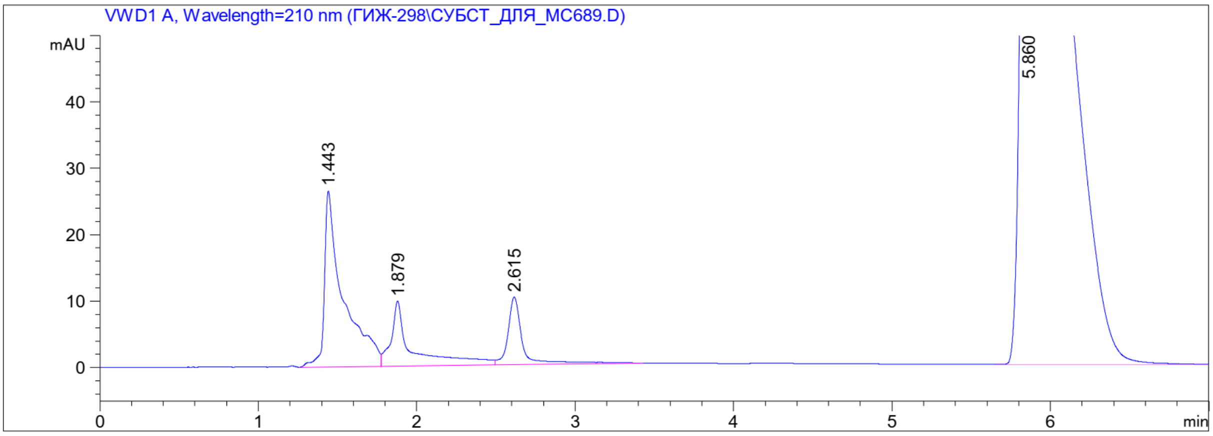

===
Отчет Относительных Площадей с Характеристиками
===

Множитель : 1.0000
Разбавление : 1.0000
Применять Множитель & Разбавление к ISTD

Сигнал 1: VWD1 A, Wavelength=210 nm

RetTime [мин]	k'	Площадь [mAU*s]	Высота [mAU]	Симм.	Ширина [мин]	Тарелки	Разрешение	Селективность
1.443	-	218.50345	26.52153	0.26	0.0826	1690	-	-
1.879	-	89.55509	9.78989	0.37	0.0633	4880	3.51	1.30
2.615	-	65.49288	10.17707	0.64	0.0800	5918	6.03	1.39
5.860	-	2383.82666	136.20351	0.20	0.2744	2526	10.76	2.24

(*Z*)-phenyl(pyridin-4-yl)methanone *O*-(2-morpholinoethyl) oxime oxalate (*Z*-GIZH-298) batch 260324 spiked with *E*-isomer

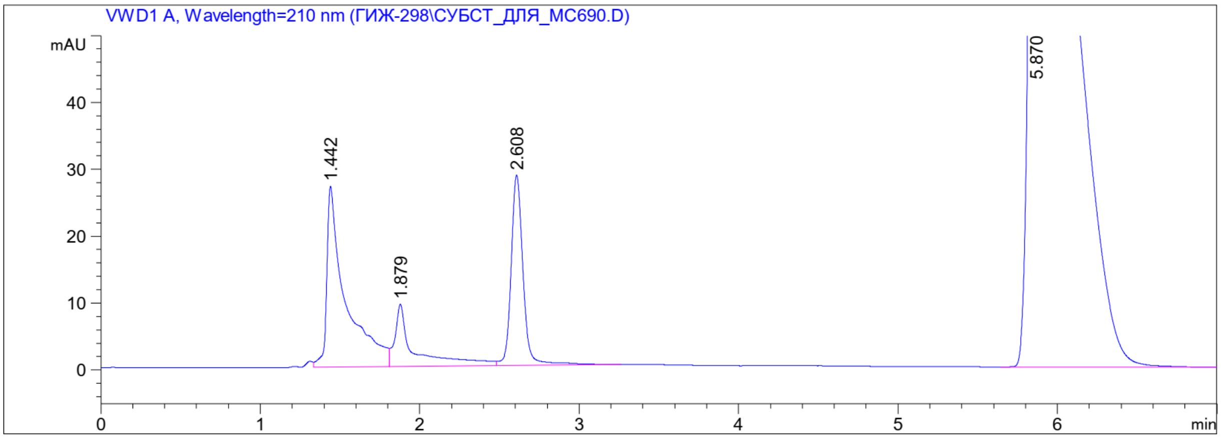

===

Отчет Относительных Площадей с Характеристиками

===

Множитель : 1.0000
Разбавление : 1.0000
Применять Множитель & Разбавление к ISTD

Сигнал 1: VWD1 A, Wavelength=210 nm

RetTime [мин]	k'	Площадь [mAU*s]	Высота [mAU]	Симм.	Ширина [мин]	Тарелки	Разрешение	Селективность
1.442	-	220.42218	27.04058	0.24	0.0817	1728	-	-
1.879	-	77.40013	9.28560	0.33	0.0633	4874	3.54	1.30
2.608	-	147.86598	28.44432	0.78	0.0766	6433	6.12	1.39
5.870	-	2242.69019	130.88702	0.21	0.2690	2637	11.09	2.25
